# Integration of Immunometabolic Composite Indices and Machine Learning for Diabetic Retinopathy Risk Stratification: Insights from NHANES 2011 – 2020

**DOI:** 10.1016/j.xops.2025.100854

**Published:** 2025-06-16

**Authors:** Cui Xuehao, Wen Dejia, Li Xiaorong

**Affiliations:** 1 John Van Geest Centre for Brain Repair and MRC Mitochondrial Biology Unit, Department of Clinical Neurosciences, University of Cambridge, Cambridge, UK; 2 Cambridge Eye Unit, Addenbrooke's Hospital, Cambridge University Hospitals, Cambridge, UK; 3 Eye Institute and School of Optometry, Tianjin Medical University Eye Hospital, Tianjin, China; 4 Tianjin Key Laboratory of Retinal Functions and Diseases, Tianjin, China; 5 Tianjin Branch of National Clinical Research Center for Ocular Disease, Tianjin, China

**Keywords:** Diabetic retinopathy, Immunometabolic indices, NHANES, Bayesian kernel machine regression, Risk stratification

## Abstract

**Objective:**

This study aimed to investigate the association between immunometabolic composite indices and diabetic retinopathy (DR) and to develop predictive models using machine learning (ML) techniques to improve early detection and risk stratification for DR.

**Design:**

A cross-sectional study.

**Subjects and Controls:**

Data from the National Health and Nutrition Examination Survey 2011–2020 were analyzed, involving 8249 participants categorized into healthy controls (n = 6830), diabetes without retinopathy (n = 918), and DR (n = 501).

**Methods:**

Immunometabolic indices reflecting insulin resistance, inflammation, and lipid metabolism were evaluated. Multivariate logistic regression models assessed associations with DR, and Bayesian kernel machine regression analyzed nonlinear interactions. Eight ML models, including ensemble methods, were developed to predict DR risk, with feature importance determined by SHapley Additive exPlanations.

**Main Outcome Measures:**

The primary outcome was DR status, classified according to the ETDRS criteria from fundus photography.

**Results:**

Key immunometabolic indices, notably Frailty Index (FRAILTY) and fasting serum insulin (FSI), were significantly associated with increased DR risk, whereas the metabolic score for insulin resistance (METS) showed a protective effect. Bayesian kernel machine regression highlighted complex interactions among indices. Machine learning models achieved high predictive accuracy, particularly XGBoost and LightGBM (area under the curve > 0.9). SHapley Additive exPlanations analyses identified FRAILTY, FSI, and METS as the most influential predictors.

**Conclusions:**

Immunometabolic dysregulation significantly contributes to DR progression beyond traditional risk factors such as hyperglycemia alone. Incorporating immunometabolic indices into predictive models substantially enhances DR risk stratification, facilitating personalized screening and intervention strategies. Machine learning approaches effectively identify high-risk individuals, underscoring their utility in clinical practice for early DR detection and targeted preventive care.

**Financial Disclosure(s):**

The author(s) have no proprietary or commercial interest in any materials discussed in this article.

Diabetic retinopathy (DR) is a progressive microvascular complication of diabetes mellitus (DM) and one of the leading causes of vision impairment and blindness worldwide.^1^, ^2^, ^3^ It arises from prolonged hyperglycemia, which induces endothelial dysfunction, increased vascular permeability, capillary occlusion, and ischemic damage in the retina.^4^, ^5^, ^6^ The underlying mechanisms involve oxidative stress, chronic inflammation, and the accumulation of advanced glycation end-products, leading to the breakdown of the blood–retinal barrier.^7^, ^8^, ^9^ As DR progresses from nonproliferative DR to proliferative DR, the development of neovascularization, vitreous hemorrhage, and retinal detachment can result in severe visual loss.^10^ Despite advancements in screening and treatment, DR remains a significant global health burden, exacerbated by the increasing prevalence of diabetes.^11^ Diabetes mellitus is a rapidly growing global epidemic, with its prevalence increasing at an alarming rate.^12^ According to recent estimates, hundreds of millions of people worldwide are living with diabetes, and this number is projected to rise significantly in the coming decades. However, not all diabetic patients develop DR.^13^ Studies suggest that approximately 30% to 40% of people with diabetes develop some form of DR, highlighting that DR is not an inevitable outcome of diabetes but, rather, a condition influenced by specific risk factors and pathophysiological mechanisms.^14^

Early identification of individuals at risk for DR is crucial for timely intervention and improved management.^15^ Although traditional risk factors such as glycemic control, hypertension, dyslipidemia, and diabetes duration have been extensively investigated, growing evidence highlights the importance of immune–metabolic dysregulation in DR pathogenesis.^16^^,^^17^ Metabolic and immune alterations contribute to systemic inflammation, insulin resistance, and vascular damage, all playing a role in DR progression.^18^ However, conventional risk assessments do not fully capture these complex interactions, necessitating composite indices integrating multiple physiological and biochemical parameters.

Unlike previous studies that primarily compared DR patients with healthy controls (HCs), our study takes a novel approach by comparing DR patients with those who have diabetes but without retinopathy. This distinction is essential because it allows us to isolate factors associated explicitly with DR progression rather than general metabolic dysfunction related to diabetes. Given that DR is a complication of diabetes, studying diabetic individuals without DR as a comparison group provides a more precise evaluation of disease-specific risk factors. This approach improves the early diagnosis and prediction of DR within the diabetic population, offering more targeted strategies for prevention and intervention. Moreover, traditional regression-based analyses often fail to account for the complex and nonlinear interactions between multiple biomarkers.^19^ In contrast, machine learning (ML) techniques provide a powerful approach to handling high-dimensional data, enabling a more robust assessment of DR risk factors.^20^ This study utilizes data from the National Health and Nutrition Examination Survey (NHANES) 2011–2020 to explore the association between immune-metabolic composite indices and DR. By combining statistical modeling with advanced ML techniques, we aim to enhance DR risk stratification and improve predictive models for early detection and prevention.

A distinctive feature of this study is the inclusion of a broad range of immunometabolic composite indices (IMCIs), offering a holistic view of metabolic dysfunction and immune-related alterations. These indices reflect the interplay between insulin resistance, lipid metabolism, inflammation, and vascular function. By leveraging these indices, our study seeks to provide a deeper understanding of how systemic metabolic and immune dysregulation contribute to DR progression, ultimately supporting the development of more personalized screening and prevention strategies.

## Methods

### Study Design and Data Source

This study is a cross-sectional analysis utilizing data from the NHANES 2011–2020. The NHANES is a nationally representative survey conducted by the Centers for Disease Control and Prevention that collects comprehensive health, demographic, and nutritional data from the U.S. population using a stratified, multistage probability sampling design. Individuals were classified into 3 groups: HCs (n = 6830), diabetes without retinopathy (DM, n = 918), and DR (n = 501). Diabetes was determined by self-reported diagnosis, physician diagnosis, or HbA1c ≥ 6.5% (Fig. S12, available at www.ophthalmologyscience.org). Diabetic retinopathy was ascertained in the NHANES dataset based on fundus photography assessments performed using standardized protocols across survey cycles. Specifically, DR status was determined according to the ETDRS grading criteria. Due to variations in photographic protocols across NHANES cycles, harmonization procedures were implemented to ensure consistency in DR classification throughout the analysis. Participants with missing demographic, metabolic, or clinical data were excluded from the analysis. The data cleaning process included several steps. First, inconsistencies and implausible values were identified and corrected. Missing values were addressed using multiple imputation techniques to minimize bias. Continuous variables were assessed for normality, and skewed distributions were log-transformed where appropriate. Duplicate records were removed, and data integrity checks ensured consistency across survey cycles.

### Study Variables

A wide range of demographic, metabolic, immunologic, and lifestyle-related variables were included to analyze DR risk factors comprehensively. Demographic variables included gender (male and female), age, and race (Mexican American, Other Hispanic, Non-Hispanic White, Non-Hispanic Black, and other race). Socioeconomic factors such as education level (less than ninth grade, 9–11th grade, high school, some college, and college or above) were also included. Anthropometric and lifestyle variables included body mass index (BMI), waist circumference, alcohol consumption, sleep duration, and hypertension status (yes/no). These factors contribute to metabolic disturbances and systemic inflammation, which may influence DR development.

### IMCIs

A key focus of this study was the evaluation of IMCIs, which integrates metabolic and immune-related parameters to provide a more comprehensive assessment of DR risk factors (Fig. S2, available at www.ophthalmologyscience.org). These indices reflect complex biological interactions involving systemic inflammation, insulin resistance, lipid metabolism, and vascular function. The following indices were included:

Body fat percentage: Represents adiposity levels and potential metabolic dysfunction.^21^

The body roundness index measures body shape and obesity-related health risks.^22^

Cardiometabolic disease assessment index: A composite score evaluating cardiometabolic disease risk.^23^

Dietary inflammatory index: Quantifies diet-related inflammation.^24^

Diet index: Dietary index for gut microbiota.^25^

Frailty Index (FRAILTY): A multidimensional measure of physiological vulnerability.^26^

Fasting serum insulin (FSI) index: Reflects insulin secretion capacity.^27^

Healthy Eating Index: Evaluates overall dietary quality.^28^

Homeostatic model assessment of insulin resistance (HOMA-IR) and homeostatic model assessment of beta-cell function: Estimates pancreatic beta-cell function and insulin resistance.^29^^,^^30^

Lipid accumulation product (LAP): A marker of visceral adiposity.^31^

Life’s Simple 7: A cardiovascular health metric.^32^

Metabolic Score for Insulin Resistance: A refined indicator of metabolic severity.^33^

Aggregate index of systemic inflammation: Comprehensively evaluate the systemic inflammatory condition by analyzing complete blood counts.^34^

Non–high-density lipoprotein cholesterol to high-density lipoprotein cholesterol ratio: A lipid-based marker that reflects the balance between atherogenic (bad) and protective (good) cholesterol.^35^

Oxidative Balance Score: Integrates multiple oxidative balance scores.^36^

Systemic immune-inflammation index: A biomarker of immune system activation.^37^

Triglyceride–glucose index (TYG): A surrogate marker for insulin resistance.^38^

Visceral adiposity index: Evaluates visceral fat-related metabolic risk.^39^

These indices were derived using established formulas incorporating anthropometric, biochemical, and clinical markers. Their inclusion allows for a refined characterization of metabolic and immune dysregulation in DR.

### Multivariate Regression Analysis

Multivariate logistic regression models assessed the relationship between IMCIs and DR.^40^ The first model examined the crude association between each index and DR without adjustments. The second model controlled for key confounders, including age, sex, race, BMI, hypertension, and other metabolic factors, to isolate the independent contribution of each index. Considering the complex stratified sampling design of the NHANES database, we acknowledge the importance of incorporating sampling weights in our analysis. However, in this study, we aimed primarily at exploring mechanistic associations rather than estimating national prevalence rates. Thus, sampling weights were not applied, but we recognize that future population-level inference studies should employ weighted analyses.

Beyond the general associations, 6 indices that showed the most significant differences between the DM and DR groups were selected for further exploration. Both linear and nonlinear relationships between these indices and DR were examined. Generalized additive models and restricted cubic splines were used to evaluate nonlinear trends and potential threshold effects. Interaction terms were incorporated into the models to test potential modifying effects by demographic and clinical variables.

### Bayesian Kernel Machine Regression

Bayesian kernel machine regression (BKMR) was applied to investigate further the relationships and interactions between metabolic indices.^41^ Bayesian kernel machine regression is a flexible modeling approach that allows for the simultaneous evaluation of multiple correlated biomarkers while accounting for nonlinear and interactive effects. For the BKMR model, we conducted Markov Chain Monte Carlo sampling with a total of 10 000 iterations following an initial burn-in of 5000 iterations, which provided sufficient convergence according to trace plots and Gelman-Rubin diagnostics. This method assessed the joint effect of the 6 most significant immunometabolic indices on DR risk, identified potential interactions among the indices and their contribution to DR, and evaluated the overall risk profile by integrating high-dimensional immunometabolic data. Posterior inclusion probabilities were computed to rank the relative importance of each variable. Sensitivity analyses were conducted by varying prior distributions to test the robustness of the findings. Partial dependence plots were generated to visualize how changes in specific indices influenced DR risk in the presence of other correlated markers. We employed Spearman correlation coefficients to analyze the correlation between immunometabolic indices and traditional risk factors because of the non-normal distribution and ordinal nature of several immunometabolic variables, making the Spearman method more robust to violations of normality assumptions.

### ML Analysis

Eight supervised ML algorithms were implemented to explore the predictive power of immunometabolic indices further to classify participants into HC, DM, and DR groups. These models included decision tree, elastic net, random forest, XGBoost, LightGBM, support vector machine, multinomial logistic regression, and multilayer perceptron.^42^ The models were trained using a cross-validation strategy to optimize hyperparameters and prevent overfitting. Performance metrics included accuracy, precision, recall, and the F1-score. Receiver operating characteristic curves were generated, and the area under the curve (AUC) was computed for each model to assess discrimination ability.

SHapley Additive exPlanations analysis was conducted^24^ to interpret the results. SHapley Additive exPlanations values quantified the contribution of each immunometabolic index to DR prediction, providing insights into which features most influenced model outputs. Partial dependence plots and feature importance rankings were used to enhance the interpretability of ML findings. By integrating traditional statistical approaches with advanced ML techniques, this study comprehensively evaluates immunometabolic indices in DR risk stratification, improving early detection and prevention strategies.

### Ethics Approval and Consent to Participate

This study utilized publicly available data from the NHANES. The NHANES was approved by the National Center for Health Statistics Research Ethics Review Board, and written informed consent was obtained from all participants at the time of data collection. Therefore, additional ethics approval was not required for this secondary analysis. This research adhered strictly to the tenets of the Declaration of Helsinki.

## Results

### Baseline Characteristics

Table 1 presents the baseline characteristics of the study population, including HCs (n = 6830), diabetes without retinopathy (DM, n = 918), and DR (n = 501). Multiple testing was corrected using the false discovery rate method. Significant differences were observed across demographic, metabolic, and immunometabolic indices. Significant demographic differences were observed among groups, with HC participants younger than DM and DR individuals (*P* < 0.001). The proportion of men was slightly higher in the DM and DR groups. Racial composition varied, with non-Hispanic Whites being more prevalent in HC, whereas non-Hispanic Black and Hispanic individuals were more common in the DM and DR groups. Diabetic retinopathy participants had lower education levels, with fewer attaining college education and more having less than a high school education compared with HC (*P* < 0.001). Metabolic risk factors were significantly elevated in DM and DR groups compared with HC, with the highest BMI and waist circumference observed in DM, followed by DR. Hypertension prevalence also increased progressively from HC to DM and DR (*P* < 0.001).

**Table 1:** The Baseline Information of the HC, DM, and DR Cohorts

Phenotype	HC	DM	DR	FDR-*P*_(DR/DM)_	FDR-*P*_(DR/HC_)
N	6830	918	501		
Gender (%)				0.738	0.206
Male	3566 (52.2)	529 (57.6)	281 (56.1)		
Female	3264 (47.8)	389 (42.4)	220 (43.9)		
Age	47.38 (17.12)	61.10 (12.62)	62.14 (11.92)	0.373	<0.001
Race (%)				**0.024**	<0.001
Mexican American	867 (12.7)	139 (15.1)	79 (15.8)		
Other Hispanic	704 (10.3)	103 (11.2)	73 (14.6)		
Non-Hispanic White	2893 (42.4)	332 (36.2)	141 (28.1)		
Non-Hispanic Black	1446 (21.2)	250 (27.2)	132 (26.3)		
Other race	920 (13.5)	94 (10.2)	76 (15.2)		
EDU (%)				0.397	<0.001
Less than 9th grade	402 (5.9)	107 (11.7)	72 (14.4)		
9–11th grade	806 (11.8)	131 (14.3)	85 (17.0)		
High school grade	1534 (22.5)	213 (23.2)	121 (24.2)		
Some college	2218 (32.5)	295 (32.1)	143 (28.5)		
College or above	1870 (27.4)	172 (18.7)	80 (16.0)		
BMI	28.89 (6.86)	32.71 (7.55)	32.20 (7.34)	0.410	<0.001
WAIST	98.51 (16.35)	110.74 (16.70)	109.50 (16.88)	0.397	<0.001
ALCOHOL	4.36 (19.25)	3.33 (4.84)	4.95 (44.62)	0.414	0.645
HBP (%)				0.410	<0.001
Yes	2144 (31.4)	625 (68.1)	358 (71.5)		
No	4686 (68.6)	293 (31.9)	143 (28.5)		
SLEEP	7.24 (1.50)	7.32 (3.44)	7.47 (1.86)	0.450	0.008
ABSI	0.08 (0.00)	0.08 (0.00)	0.08 (0.00)	0.373	<0.001
BFP	34.35 (10.09)	39.33 (9.29)	39.11 (9.55)	0.775	<0.001
BRI	5.32 (2.32)	7.07 (2.57)	7.08 (2.61)	0.943	<0.001
CDAI	0.65 (4.13)	0.31 (3.62)	−0.21 (3.50)	0.054	<0.001
DII	1.50 (1.90)	1.65 (1.84)	1.81 (1.76)	0.357	<0.001
DI_GM	4.57 (1.60)	4.36 (1.65)	4.47 (1.58)	0.414	0.266
**DASH**	2.25 (1.51)	2.11 (1.49)	2.38 (1.51)	**0.024**	0.155
**FRAILTY**	0.14 (0.09)	0.25 (0.10)	0.31 (0.12)	**<0.001**	<0.001
**FSI**	−1.39 (1.63)	−0.08 (1.72)	0.52 (1.35)	**<0.001**	<0.001
HEI2015	50.00 (14.01)	50.38 (13.28)	50.18 (13.25)	0.856	0.787
HOMA_IR	3.23 (3.52)	9.64 (18.90)	7.90 (16.65)	0.336	<0.001
HOMA_BETA	97.22 (1628.77)	118.42 (271.51)	156.10 (300.10)	0.077	0.559
**LAP**	49.57 (47.55)	81.64 (77.93)	63.70 (46.68)	**<0.001**	<0.001
**LS7**	8.44 (2.29)	5.78 (1.79)	5.48 (1.85)	**0.024**	<0.001
**METS**	42.40 (12.23)	52.26 (14.06)	47.53 (9.99)	**<0.001**	<0.001
AISI	3838.17 (1794.02)	4089.60 (2219.10)	4204.65 (1993.10)	0.447	<0.001
NHHR	2.80 (1.10)	2.85 (0.98)	2.92 (1.03)	0.410	0.096
**OBS**	19.99 (5.72)	19.11 (5.37)	17.97 (6.10)	**<0.001**	<0.001
SII	493.28 (301.30)	554.87 (368.91)	574.48 (361.30)	0.447	<0.001
**TYG**	8.46 (0.63)	9.11 (0.76)	8.80 (0.61)	**<0.001**	<0.001
VAI	1.73 (1.89)	2.54 (2.66)	2.53 (1.75)	0.943	<0.001

ABSI = a body shape index; AISI = aggregate index of systemic inflammation; BFP = body fat percentage; BMI = body mass index; BRI = body roundness index; CDAI = cardiometabolic disease assessment index; DASH = Dietary Approach to Systolic Hypertension; DII = dietary inflammatory index; DI_GM = dietary index for gut microbiota; DM = diabetes mellitus; DR = diabetic retinopathy; EDU = education; FDR = false discovery rate; FRAILTY = Frailty Index; FSI = fasting serum insulin; HBP = high blood pressure; HC = healthy control; HEI2015 = Healthy Eating Index; HOMA_BETA = homeostatic model assessment of beta-cell function; HOMA_IR = homeostatic model assessment of insulin resistance; LAP = lipid accumulation product; LS7 = Life’s Simple 7; METS = metabolic score for insulin resistance; NHHR = non–high-density lipoprotein cholesterol to high-density lipoprotein cholesterol ratio; OBS = oxidative balance score; SII = systemic immune-inflammation index; VAI = visceral adiposity index; TYG = triglyceride–glucose index.

Bold indicates *P* < 0.05.

Several immunometabolic indices were significantly different among the 3 groups. The FRAILTY increased progressively from HC (0.14 ± 0.09) to DM (0.25 ± 0.10) and DR (0.31 ± 0.12) (*P* < 0.001). Fasting serum insulin was also significantly higher in DR (0.52 ± 1.35) than in DM (−0.08 ± 1.72) and HC (−1.39 ± 1.63) (*P* < 0.001). Lipid accumulation product, metabolic score for insulin resistance (METS), and TYG were significantly elevated in DR compared with DM and HC, suggesting their role in DR pathogenesis. Nutritional and inflammatory markers also showed notable variations. The dietary inflammatory index was highest in DR (1.81 ± 1.76) compared with DM (1.65 ± 1.84) and HC (1.50 ± 1.90) (*P* < 0.001), implying a link between diet-related inflammation and DR development.

### Multivariate Regression Analysis of IMCIs

**Figure 1:**
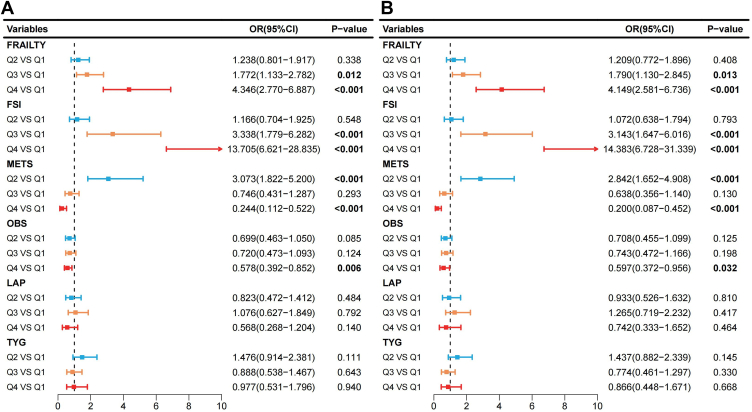
Odds ratios for different variables. This figure presents forest plots showing the ORs and 95% CIs for various risk factors across different quartiles (Q2, Q3, Q4 vs. Q1). Panels **(A)** and **(B)** compare the associations of Frailty, FSI, METS, OBS, LAP, and TYG with the outcome of interest. Significant associations (*P* < 0.05) are highlighted in bold. The horizontal lines represent CIs, and colors indicate different quartiles. CI = confidence interval; FSI = fasting serum insulin; LAP = lipid accumulation product; OBS = oxidative balance score; OR = odds ratio; TYG = triglyceride–glucose index.

Figure 1 presents the multivariate regression analysis of the 6 most significant IMCIs stratified by quartiles. Figure 1A shows the unadjusted model, whereas Figure 1B illustrates the fully adjusted model, controlling for potential confounders, including age, sex, race, BMI, hypertension, and other metabolic factors. In the unadjusted model, FRAILTY, FSI, and METS are strongly positively associated with DR risk. Individuals in the highest quartile (Q4) of FRAILTY had a significantly higher risk of DR (odds ratio [OR] = 4.346, 95% confidence interval [CI]: 2.770–6.887, *P* < 0.001). Similarly, higher FSI levels were strongly associated with increased DR risk (Q4 vs. Q1: OR = 13.705, 95% CI: 6.621–28.835, *P* < 0.001). The metabolic score for insulin resistance showed an inverse association, with Q4 individuals having a lower DR risk (OR = 0.244, 95% CI: 0.112–0.522, *P* < 0.001). Oxidative balance score also demonstrated a protective effect (OR = 0.578, 95% CI: 0.392–0.852, *P* = 0.006), whereas LAP and TYG showed no significant associations.

After adjusting for confounding variables, The Frailty Index, FSI, and METS remained significantly associated with DR risk. The fully adjusted model confirmed that individuals in the highest quartile of FRAILTY had a significantly increased risk of DR (OR = 4.149, 95% CI: 2.581–6.736, *P* < 0.001). Fasting serum insulin remained a strong predictor of DR, with an even higher OR in Q4 (OR = 14.383, 95% CI: 6.728–31.339, *P* < 0.001). With DR, METS retained its inverse association, with Q4 showing a lower risk (OR = 0.200, 95% CI: 0.087–0.452, *P* < 0.001). Oxidative balance score continued to exhibit a significant protective effect in Q4 (OR = 0.597, 95% CI: 0.372–0.956, *P* = 0.032), although slightly attenuated compared with the unadjusted model. Lipid accumulation product and TYG did not show significant associations with DR in the adjusted analysis.

**Figure 2:**
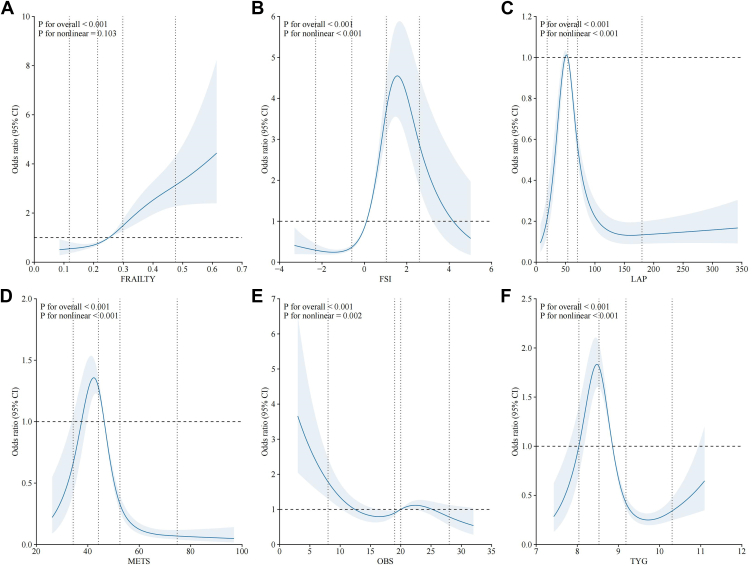
Nonlinear associations between variables and outcome. This figure illustrates the nonlinear relationships between 6 key variables (FRAILTY, FSI, METS, OBS, LAP, and TYG) and the outcome. The blue solid line represents the estimated odds ratio, whereas the shaded area indicates the 95% CI. The *P* values for overall and nonlinear effects are displayed in each panel, showing whether the association is statistically significant. CI = confidence interval; FSI = fasting serum insulin; LAP = lipid accumulation product; OBS = oxidative balance score; TYG = triglyceride–glucose index.

After adjusting for all covariates, the nonlinear relationships between the 6 most significant immunometabolic indices and DR risk were assessed. The Frailty Index (Fig 2A) showed a positive linear association with DR risk, with a steeper OR increase at higher levels, indicating a progressively higher risk as frailty worsens. Fasting serum insulin (Fig 2B) exhibited a pronounced nonlinear relationship, with a sharp rise in DR risk beyond a threshold level, suggesting that excessive FSI is strongly linked to DR development. Lipid accumulation product (Fig 2C) demonstrated an inverse U-shaped relationship with DR, in which moderate levels were associated with the lowest risk. In contrast, very high or very low levels corresponded to increased DR risk. With higher METS scores linked to a lower risk of DR, METS (Fig 2D) displayed a strong inverse association, reinforcing its protective effect. Oxidative balance score (Fig 2E) showed a relatively stable association, with a slight protective effect at moderate levels and an increased risk at extreme values. Triglyceride–glucose index (Fig 2F) revealed a bimodal pattern, with 2 peaks of increased risk at lower and higher values, indicating a complex relationship between triglyceride–glucose interactions and DR.

### Interaction Effects and Combined Influence of Immunometabolic Indices Using BKMR

**Figure 3:**
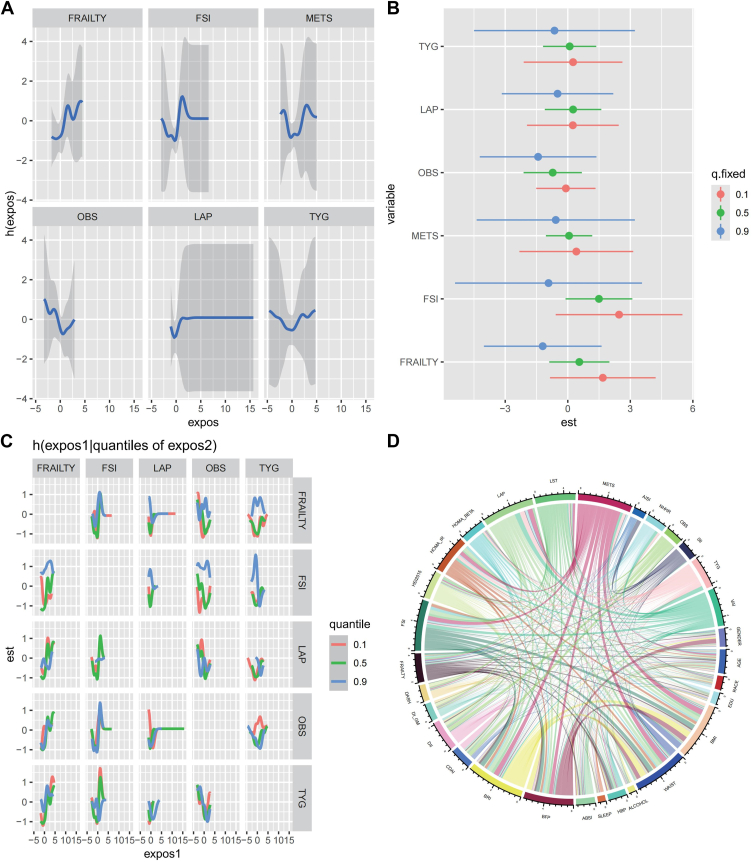
Interaction and quantile regression analysis. This figure explores the interaction effects between different variables using quantile regression and network visualization. Panel **(A)**: Smoothing splines depicting the associations between exposure variables and the outcome. Panel **(B)**: Quantile regression estimates for different variables, where points represent estimates at different quantiles. Panel **(C)**: Interaction effects between pairs of exposures, with color-coded quantiles. Panel **(D)**: Chord diagram visualizing complex interrelationships among the studied variables. FSI = fasting serum insulin; LAP = lipid accumulation product; OBS = oxidative balance score; TYG = triglyceride–glucose index.

Bayesian kernel machine regression was employed to evaluate the combined effects and interactions among immunometabolic indices on DR risk (Figs 3A, S1, available at www.ophthalmologyscience.org). This flexible modeling approach allowed for the simultaneous assessment of multiple correlated indices, capturing nonlinear relationships and potential interaction effects. The Frailty Index and FSI were strongly associated with DR, both independently and in combination, suggesting that increased frailty and insulin resistance exacerbate disease progression. The metabolic score for insulin resistance consistently showed a protective effect, reinforcing its role in mitigating DR risk in the DM population. The BKMR model demonstrated that when considering joint effects, the overall contribution of these indices to DR risk remained significant (Fig 3B). This approach provided insights into how specific index combinations influence disease risk beyond their individual effects. The interactions between FRAILTY, FSI, METS, and LAP suggested potential synergistic effects, in which certain index combinations amplified or mitigated DR susceptibility. Oxidative balance score and LAP exhibited moderate associations, with their influence on DR risk varying across different exposure levels. Triglyceride–glucose index showed inconsistent effects, indicating a more nuanced relationship with DR development (Fig 3C, S1). Correlation analysis further highlighted strong interconnections between immunometabolic indices and other metabolic parameters, emphasizing their shared influence on DR progression (Fig 3D). The Frailty Index and FSI were closely linked to systemic inflammation markers, whereas METS exhibited stronger lipid and glucose metabolism associations. These findings underscore the need to consider the joint impact of metabolic and immune dysregulation in DR risk assessment, supporting a more integrated approach to early intervention strategies.

### Correlation between Immunometabolic Indices and Traditional Risk Factors

The correlation analysis revealed distinct relationships between immunometabolic indices and traditional risk factors (Fig S11, available at www.ophthalmologyscience.org). FRAILTY_SCORE showed a moderate positive correlation with age (*r* = 0.40), suggesting that frailty increases with advancing age. It exhibited weaker correlations with obesity-related indicators (BMI, *r* = 0.25; waist circumference, *r* = 0.30) and insulin resistance markers (HOMA-IR, *r* = 0.18; TYG, *r* = 0.19). In contrast, FSI was strongly correlated with obesity measures, BMI (*r* = 0.82), and waist circumference (*r* = 0.83), indicating a close association with adiposity. Additionally, FSI demonstrated moderate-to-strong associations with insulin resistance indices (HOMA-IR, *r* = 0.32; TYG, *r* = 0.58) but only a weak correlation with age (*r* = 0.23). Similarly, metabolic score for insulin resistance exhibited very strong correlations with BMI (*r* = 0.91) and waist circumference (*r* = 0.87), as well as moderate correlations with insulin resistance measures (HOMA-IR, *r* = 0.35; TYG, *r* = 0.51). Notably, metabolic score for insulin resistance had negligible association with age (*r* = 0.04), implying that it predominantly reflects metabolic and adiposity-driven factors rather than aging itself. Overall, these findings highlight that FRAILTY primarily reflects age-related physiological vulnerability, whereas FSI and metabolic score for insulin resistance predominantly captures metabolic dysfunction related to obesity and insulin resistance.

### ML-Based Prediction of DR

**Figure 4:**
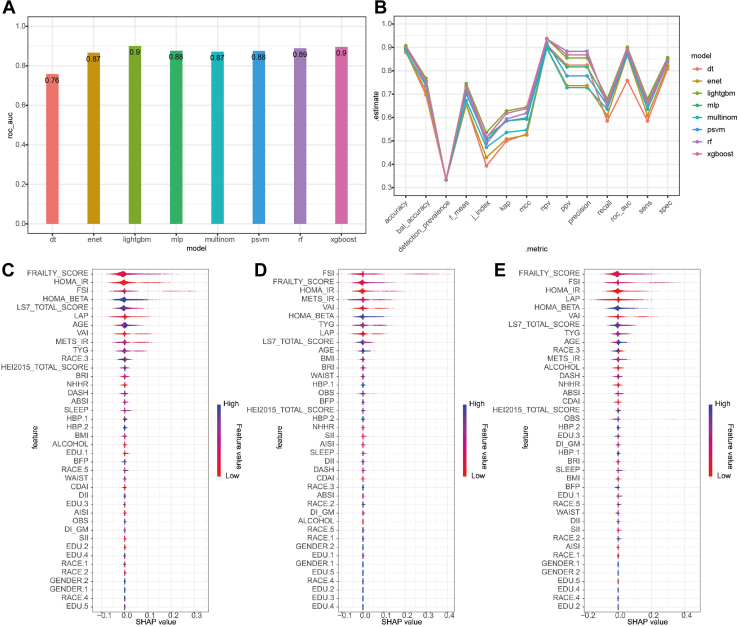
Machine learning model performance and feature importance. This figure evaluates the predictive power of various machine learning models in classifying the outcome based on different risk factors. Panel **(A)**: Area under the receiver operating characteristic curve for different models. Panel **(B)**: Comparison of model estimates across features. Panels **(C–E)**: SHapley Additive exPlanations values showing the most important predictive features for XGBoost, RF, and LightGBM models, with red indicating high feature values and blue indicating low feature values. AISI = aggregate index of systemic inflammation; BFP = body fat percentage; BMI = body mass index; BRI = body roundness index; CDAI = cardiometabolic disease assessment index; DI_GM = dietary index for gut microbiota; DII = dietary inflammatory index; FSI = fasting serum insulin; LAP = lipid accumulation product; HOMA_BETA = homeostatic model assessment of beta-cell function; HOMA_IR = homeostatic model assessment of insulin resistance; LS7 = Life’s Simple 7; NHHR = non–high-density lipoprotein cholesterol to high-density lipoprotein cholesterol ratio; OBS = oxidative balance score; SII = systemic immune-inflammation index; RF = random forest; VAI = visceral adiposity index; TYG = triglyceride–glucose index.

To evaluate the predictive value of immunometabolic indices for DR, 8 ML models were trained using all demographic, metabolic, and clinical variables (Figs 4, S3–S10, Tables S1–S8, available at www.ophthalmologyscience.org). Models were optimized through cross-validation, and their predictive performances were assessed using the AUC. The results showed that ensemble learning models, particularly XGBoost and LightGBM, achieved the highest predictive accuracy (AUC > 0.9), indicating their robustness in distinguishing between DR, DM, and HC. Traditional methods, such as decision tree, showed the lowest performance (AUC = 0.76), highlighting the limitations of more straightforward classification techniques (Fig 4A). The ensemble models exhibited superior stability and generalizability, making them more suitable for DR prediction in clinical settings. Further comparison of model performance (Fig 4B) revealed that ensemble learning models consistently outperformed traditional statistical approaches, demonstrating their ability to capture complex interactions among metabolic and immune-related factors. Machine learning allows for higher classification accuracy and better feature integration, reducing prediction bias in distinguishing DM from DR.

**Figure 5:**
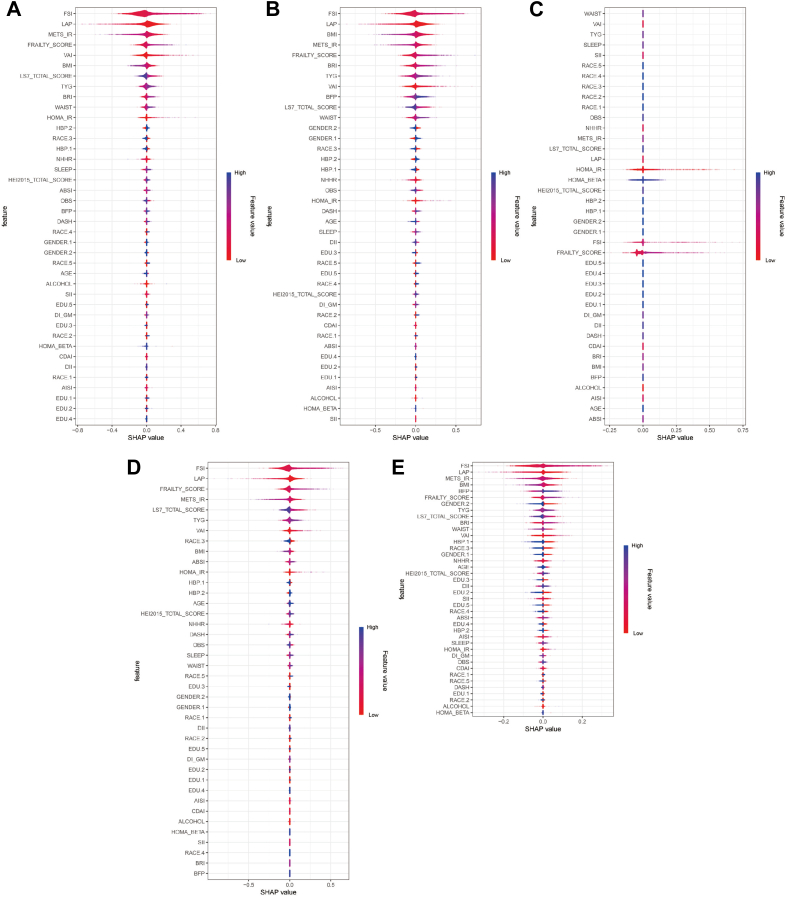
SHapley Additive exPlanations value analysis for different models. This figure further explores feature importance across multiple models, including SVM, multinomial, decision tree, elastic net, and MLP. Panels **(A–E)**: SHAP values for each model, showing how individual variables contribute to the model predictions. Higher absolute SHAP values indicate a stronger influence on the outcome. AISI = aggregate index of systemic inflammation; BMI = body mass index; BFP = body fat percentage; BRI = body roundness index; CDAI = cardiometabolic disease assessment index; DI_GM = dietary index for gut microbiota; DII = dietary inflammatory index; FSI = fasting serum insulin; HOMA_BETA = homeostatic model assessment of beta-cell function; HOMA_IR = homeostatic model assessment of insulin resistance; LAP = lipid accumulation product; LS7 = Life’s Simple 7; OBS = oxidative balance score; NHHR = non–high-density lipoprotein cholesterol to high-density lipoprotein cholesterol ratio; MLP = multilayer perceptron; SHAP = SHapley Additive exPlanations; SVM = support vector machine; SII = systemic immune-inflammation index; VAI = visceral adiposity index; TYG = triglyceride–glucose index.

SHapley Additive exPlanations analysis was conducted to enhance interpretability, identifying the most influential features contributing to DR prediction (Figs 4C–E and 5A–E). This approach quantified the impact of each variable on model predictions, enabling a more transparent understanding of the driving factors behind DR classification. SHapley Additive exPlanations analysis revealed that FRAILTY, FSI, and METS were consistently among the top predictors of DR risk across all models. Their strong predictive power underscores the critical role of metabolic and immune dysregulation in DR pathogenesis. Homeostatic model assessment of insulin resistance, Life’s Simple 7, and lipid-related indices also contributed significantly to DR classification, confirming the involvement of insulin resistance and systemic metabolic dysfunction in DR progression. Other vital contributors included HOMA-IR, Life’s Simple 7 total score, and lipid-related indices, indicating that metabolic and immune dysfunction collectively drive DR development. Machine learning models, particularly ensemble methods, demonstrated strong predictive power in DR risk assessment. Their ability to integrate immunometabolic indices improves diagnostic accuracy, facilitating targeted screening and early intervention in diabetic populations.

## Discussion

Our study systematically evaluated immunometabolic indices in distinguishing DR from diabetes without retinopathy. We identified FRAILTY, FSI, and METS as key predictors of DR, with FRAILTY and FSI positively associated with increased DR risk, whereas METS exhibited a protective effect. Bayesian kernel machine regression revealed complex interdependencies between these indices, highlighting the role of metabolic and immune dysregulation in DR pathogenesis. Furthermore, ML models incorporating immunometabolic indices significantly improved DR risk prediction, with XGBoost and LightGBM demonstrating superior performance. Our study systematically explored immunometabolic indices distinguishing DR from diabetes without retinopathy, revealing critical insights into these indices' pathophysiology and predictive potential.

Frailty Index, which reflects systemic physiological vulnerability, is increasingly recognized as a predictor of adverse health outcomes, including diabetes-related complications.^43^ Chronic systemic inflammation, oxidative stress, and endothelial dysfunction may explain its strong association with DR.^44^ Frailty is characterized by increased circulating inflammatory cytokines, which can exacerbate retinal inflammation, promote vascular leakage, and accelerate neurodegeneration in the retina.^45^ The observed nonlinear association suggests that frailty drastically increases DR risk beyond a specific threshold, reinforcing the need for early metabolic intervention.^46^ Fasting serum insulin, a marker of insulin resistance, was one of our study's strongest predictors of DR.^47^ Chronic hyperinsulinemia and insulin resistance contribute to DR through multiple mechanisms, including endothelial dysfunction, increased reactive oxygen species production, and dysregulated angiogenesis.^48^^,^^49^ Elevated insulin levels promote VEGF-mediated neovascularization, a hallmark of proliferative DR. The threshold-dependent increase in DR risk with rising FSI levels supports the notion that insulin resistance is a key driver of disease progression, independent of glycemic control.^50^ The metabolic score for insulin resistance, a composite measure of insulin resistance, lipid metabolism, and systemic inflammation, demonstrated an inverse association with DR in the DM population.^51^ This protective effect suggests that individuals with better metabolic efficiency are less prone to developing microvascular complications. Improved insulin sensitivity may reduce retinal ischemia, oxidative stress, and inflammation, thereby preserving vascular integrity and reducing DR susceptibility.^7^^,^^52^ A key strength of our study is the direct comparison between DR and DM groups, revealing that systemic insulin resistance and inflammatory burden are major differentiators of DR within the diabetic population. Diabetic retinopathy patients exhibited more severe immune-metabolic dysregulation, particularly with elevated FSI and FRAILTY levels. The observed nonlinear associations suggest threshold-dependent effects, in which DR risk increases significantly only beyond certain metabolic thresholds, emphasizing the need for early intervention before these critical points are reached.

The study suggests that immunometabolic dysfunction contributes to DR through multiple interrelated pathways, including oxidative stress, chronic inflammation, endothelial dysfunction, and neurovascular degeneration.^53^ Oxidative stress is a well-established contributor to DR, primarily driven by chronic hyperglycemia and insulin resistance.^54^ The Frailty Index and FSI, linked to increased DR risk, may reflect a more significant systemic oxidative burden. Elevated insulin levels enhance mitochondrial dysfunction and reactive oxygen species production, leading to direct endothelial damage and breakdown of the blood–retinal barrier.^55^ This increases vascular permeability, microaneurysm formation, and progressive capillary dropout.^56^ Chronic inflammation further exacerbates DR by promoting retinal microvascular damage and neuroinflammation.^57^ Elevated levels of inflammatory cytokines such as tumor necrosis factor α, interleukin 6, and C-reactive protein, commonly associated with FRAILTY and insulin resistance, have been implicated in DR pathogenesis.^58^ These cytokines upregulate VEGF expression, driving pathological angiogenesis and contributing to retinal fibrosis.^59^ In addition, inflammation-mediated microglial activation disrupts the neurovascular unit, leading to retinal ganglion cell loss and visual impairment.^60^ Endothelial dysfunction represents another key pathway linking immunometabolic dysregulation to DR.^61^ Insulin resistance impairs endothelial nitric oxide production, reducing vasodilation and exacerbating capillary nonperfusion.^62^ This leads to retinal hypoxia, triggering a compensatory increase in VEGF levels and subsequent neovascularization. The observed protective effect of METS in the DM population suggests that maintaining metabolic homeostasis may help preserve endothelial function and reduce DR risk. Lipid metabolism also plays a crucial role in DR development. Triglyceride–glucose index, which reflects triglyceride–glucose interactions, exhibited a bimodal association with DR, suggesting a complex interplay between lipid dysregulation and retinal health.^63^ Although hyperlipidemia contributes to lipotoxicity and oxidative stress, moderate lipid levels may provide neuroprotective benefits by stabilizing cell membranes and reducing inflammation.^64^ This nuanced relationship warrants further investigation.

Our study demonstrated that ML models integrating IMCIs significantly improved DR risk prediction. SHapley Additive exPlanations analysis revealed that IMCIs contributed independently to DR classification beyond conventional risk factors such as glycated hemoglobin A1c and blood pressure. This suggests that metabolic and inflammatory dysregulation precede structural retinal changes, underscoring the need for early metabolic intervention. The ability of ML models to process high-dimensional data and identify nonlinear interactions highlights their potential in precision medicine, offering a more personalized approach to DR risk assessment. Our findings underscore the necessity of incorporating IMCIs into DR risk assessment. Traditional screening primarily relies on retinal imaging and glucose control, yet our study suggests that systemic metabolic and inflammatory factors contribute significantly to DR susceptibility. Identifying high-risk individuals based on immunometabolic profiles could enable earlier intervention, potentially delaying or preventing DR onset. Future research should focus on validating these findings in longitudinal cohorts to assess the temporal relationship between immunometabolic dysregulation and DR progression. Additionally, mechanistic studies are needed to elucidate the precise molecular pathways linking immunometabolic indices to retinal damage. Exploring therapeutic interventions targeting insulin resistance, inflammation, and oxidative stress may provide novel strategies for DR prevention.

### Study Strengths and Limitations

A significant strength of our study is the inclusion of a wide range of IMCIs, allowing for a comprehensive assessment of metabolic and inflammatory factors in DR. By directly comparing DR patients with diabetic individuals without retinopathy, we identified key differentiating factors that may contribute specifically to DR pathogenesis. Additionally, our integration of ML techniques enhances the predictive accuracy of these indices, demonstrating their potential utility in risk stratification beyond traditional statistical models. Bayesian kernel machine regression provided novel insights into the complex interactions among these indices, capturing nonlinear and joint effects that may otherwise be overlooked in conventional regression models.

However, several limitations should be acknowledged. First, the cross-sectional design of NHANES prevents us from establishing causal relationships between immunometabolic indices and DR progression. Longitudinal studies are needed to validate these associations over time. Second, although NHANES provides a large, nationally representative dataset, certain variables related to retinal imaging and advanced DR staging were unavailable, limiting the granularity of our analysis. Third, although ML models improved predictive performance, their application in clinical practice requires further validation and external testing across diverse populations to ensure generalizability. Finally, residual confounding from unmeasured factors cannot be ruled out despite adjusting for multiple confounders.

### Conclusion

In summary, our study highlights the critical role of immunometabolic indices in DR pathogenesis, distinguishing DR from diabetes without retinopathy. FRAILTY, FSI, and METS emerged as key predictors, revealing novel insights into the metabolic and immune mechanisms underlying DR progression. The strong predictive performance of ML models integrating these indices suggests that incorporating immunometabolic markers into clinical workflows could enhance DR screening and early intervention. These findings support a paradigm shift in DR risk assessment, emphasizing metabolic and inflammatory factors as essential contributors to disease development and progression.

## Data Availability

The data presented in this study are available in this article and supplementary materials.
