# Supplementary material for: Integration of Immunometabolic Composite Indices and Machine Learning for Diabetic Retinopathy Risk Stratification: Insights from NHANES 2011 – 2020
Source: Ophthalmol Sci. 2025 Jun 16;5(6):100854. doi: 10.1016/j.xops.2025.100854 (PMC12329596; doi:10.1016/j.xops.2025.100854)
Supplement: Figure S1 [file mmc1.pdf]

Figure S1

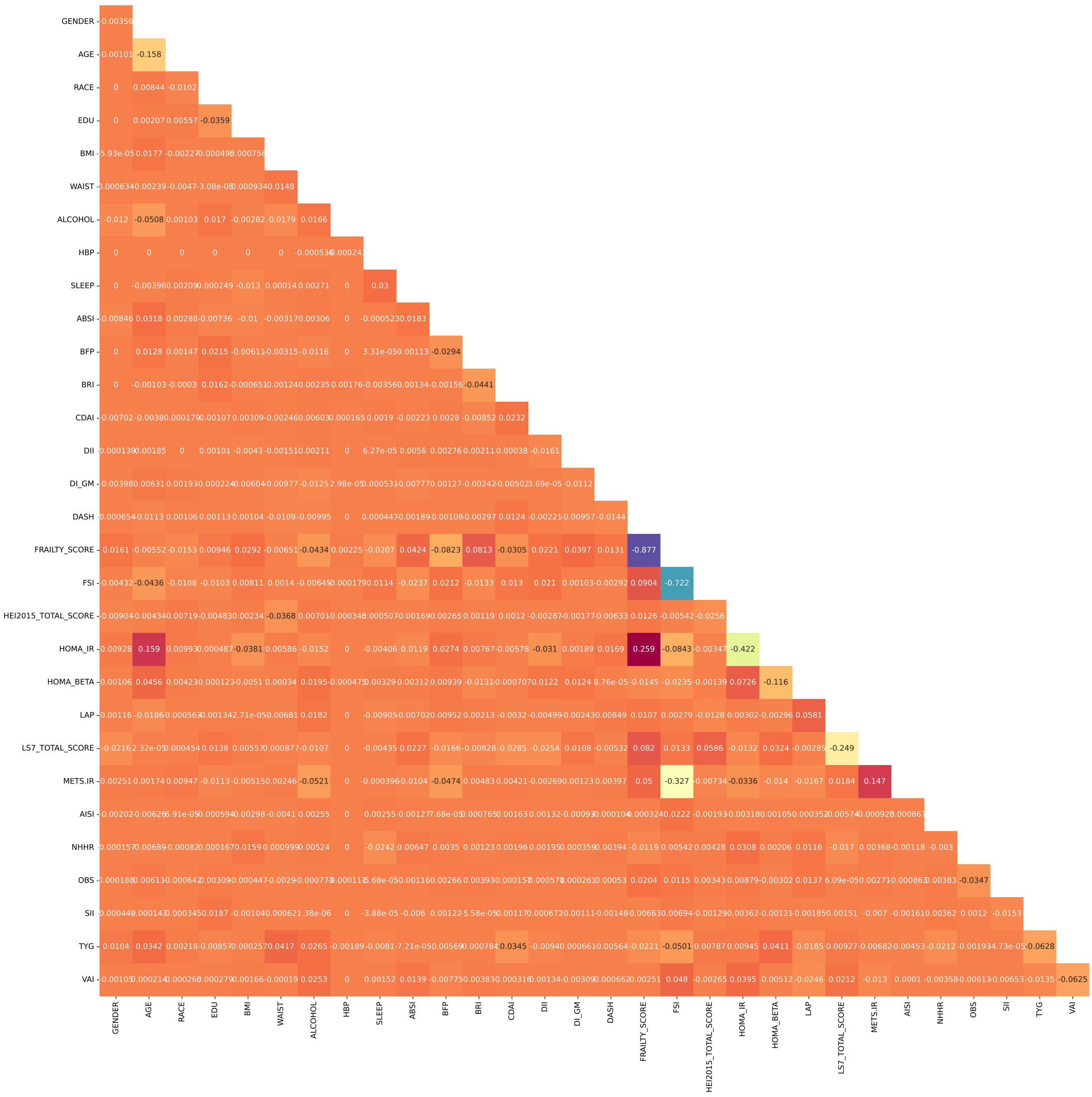

Figure S1: Correlation Heatmap

This figure presents a correlation heatmap showing the relationships between different variables. The values in each cell represent the correlation coefficient between two variables. Strong positive and negative correlations are color-coded, with deeper colors indicating stronger associations.
