# Supplementary material for: Integration of Immunometabolic Composite Indices and Machine Learning for Diabetic Retinopathy Risk Stratification: Insights from NHANES 2011 – 2020
Source: Ophthalmol Sci. 2025 Jun 16;5(6):100854. doi: 10.1016/j.xops.2025.100854 (PMC12329596; doi:10.1016/j.xops.2025.100854)
Supplement: Table S2 [file mmc3.pdf]

.metric	.estimator	.estimate	dataset	model
accuracy	multiclass	0.88393145	train	dt
kap	multiclass	0.52102268	train	dt
sens	macro	0.59114010	train	dt
spec	macro	0.81832463	train	dt
ppv	macro	0.83252828	train	dt
npv	macro	0.92578721	train	dt
mcc	multiclass	0.55082100	train	dt
j_index	macro	0.40946474	train	dt
bal_accura	macro	0.70473237	train	dt
detection_	macro	0.33333333	train	dt
precision	macro	0.83252828	train	dt
recall	macro	0.59114010	train	dt
f_meas	macro	0.66182235	train	dt
roc_auc	hand_till	0.77551480	train	dt
accuracy	multiclass	0.88269510	test	dt
kap	multiclass	0.49951630	test	dt
sens	macro	0.58534394	test	dt
spec	macro	0.80801584	test	dt
ppv	macro	0.82404908	test	dt
npv	macro	0.91027184	test	dt
mcc	multiclass	0.52911422	test	dt
j_index	macro	0.39335979	test	dt
bal_accura	macro	0.69667989	test	dt
detection_	macro	0.33333333	test	dt
precision	macro	0.82404908	test	dt
recall	macro	0.58534394	test	dt
f_meas	macro	0.65639067	test	dt
roc_auc	hand_till	0.75827587	test	dt
