# Supplementary material for: Integration of Immunometabolic Composite Indices and Machine Learning for Diabetic Retinopathy Risk Stratification: Insights from NHANES 2011 – 2020
Source: Ophthalmol Sci. 2025 Jun 16;5(6):100854. doi: 10.1016/j.xops.2025.100854 (PMC12329596; doi:10.1016/j.xops.2025.100854)
Supplement: Table S3 [file mmc4.pdf]

.metric	.estimatoı	.estimate	dataset	model
accuracy	multiclass	0.87746524	train	enet
kap	multiclass	0.50987373	train	enet
sens	macro	0.59601314	train	enet
spec	macro	0.82105420	train	enet
ppv	macro	0.75419535	train	enet
npv	macro	0.90765212	train	enet
mcc	multiclass	0.53025130	train	enet
j_index	macro	0.41706735	train	enet
bal_accura	macro	0.70853367	train	enet
detection_	macro	0.33333333	train	enet
precision	macro	0.75419535	train	enet
recall	macro	0.59601314	train	enet
f_meas	macro	0.65128607	train	enet
roc_auc	hand_till	0.87276711	train	enet
accuracy	multiclass	0.87881725	test	enet
kap	multiclass	0.50818562	test	enet
sens	macro	0.60767767	test	enet
spec	macro	0.82156853	test	enet
ppv	macro	0.73621222	test	enet
npv	macro	0.89890807	test	enet
mcc	multiclass	0.52525927	test	enet
j_index	macro	0.42924620	test	enet
bal_accura	macro	0.71462310	test	enet
detection_	macro	0.33333333	test	enet
precision	macro	0.73621222	test	enet
recall	macro	0.60767767	test	enet
f_meas	macro	0.65520097	test	enet
roc_auc	hand_till	0.86661575	test	enet
