# Supplementary material for: Integration of Immunometabolic Composite Indices and Machine Learning for Diabetic Retinopathy Risk Stratification: Insights from NHANES 2011 – 2020
Source: Ophthalmol Sci. 2025 Jun 16;5(6):100854. doi: 10.1016/j.xops.2025.100854 (PMC12329596; doi:10.1016/j.xops.2025.100854)
Supplement: Table S4 [file mmc5.pdf]

.metric	.estimator	.estimate	dataset	model
accuracy	multiclass	0.91529259	train	lightgbm
kap	multiclass	0.67219510	train	lightgbm
sens	macro	0.70190336	train	lightgbm
spec	macro	0.86988229	train	lightgbm
ppv	macro	0.88346397	train	lightgbm
npv	macro	0.95356201	train	lightgbm
mcc	multiclass	0.68915098	train	lightgbm
j_index	macro	0.57178566	train	lightgbm
bal_accura	macro	0.78589283	train	lightgbm
detection_	macro	0.33333333	train	lightgbm
precision	macro	0.88346397	train	lightgbm
recall	macro	0.70190336	train	lightgbm
f_meas	macro	0.76967214	train	lightgbm
roc_auc	hand_till	0.93631787	train	lightgbm
accuracy	multiclass	0.90693165	test	lightgbm
kap	multiclass	0.62807398	test	lightgbm
sens	macro	0.67946497	test	lightgbm
spec	macro	0.85605938	test	lightgbm
ppv	macro	0.85481793	test	lightgbm
npv	macro	0.93625055	test	lightgbm
mcc	multiclass	0.64445768	test	lightgbm
j_index	macro	0.53552436	test	lightgbm
bal_accura	macro	0.76776218	test	lightgbm
detection_	macro	0.33333333	test	lightgbm
precision	macro	0.85481793	test	lightgbm
recall	macro	0.67946497	test	lightgbm
f_meas	macro	0.74483228	test	lightgbm
roc_auc	hand_till	0.90046234	test	lightgbm
