# Supplementary material for: Integration of Immunometabolic Composite Indices and Machine Learning for Diabetic Retinopathy Risk Stratification: Insights from NHANES 2011 – 2020
Source: Ophthalmol Sci. 2025 Jun 16;5(6):100854. doi: 10.1016/j.xops.2025.100854 (PMC12329596; doi:10.1016/j.xops.2025.100854)
Supplement: Table S5 [file mmc6.pdf]

.metric	.estimator	.estimate	dataset	model
accuracy	multiclass	0.89217588	train	mlp
kap	multiclass	0.59631215	train	mlp
sens	macro	0.66421365	train	mlp
spec	macro	0.85698250	train	mlp
ppv	macro	0.82026205	train	mlp
npv	macro	0.91315629	train	mlp
mcc	multiclass	0.60504298	train	mlp
j_index	macro	0.52119616	train	mlp
bal_accura	macro	0.76059808	train	mlp
detection_	macro	0.33333333	train	mlp
precision	macro	0.82026205	train	mlp
recall	macro	0.66421365	train	mlp
f_meas	macro	0.72005419	train	mlp
roc_auc	hand_till	0.88424086	train	mlp
accuracy	multiclass	0.89190499	test	mlp
kap	multiclass	0.58559072	test	mlp
sens	macro	0.66507129	test	mlp
spec	macro	0.85116241	test	mlp
ppv	macro	0.81630411	test	mlp
npv	macro	0.90166772	test	mlp
mcc	multiclass	0.59304935	test	mlp
j_index	macro	0.51623370	test	mlp
bal_accura	macro	0.75811685	test	mlp
detection_	macro	0.33333333	test	mlp
precision	macro	0.81630411	test	mlp
recall	macro	0.66507129	test	mlp
f_meas	macro	0.72032121	test	mlp
roc_auc	hand_till	0.87620592	test	mlp
