# Supplementary material for: Integration of Immunometabolic Composite Indices and Machine Learning for Diabetic Retinopathy Risk Stratification: Insights from NHANES 2011 – 2020
Source: Ophthalmol Sci. 2025 Jun 16;5(6):100854. doi: 10.1016/j.xops.2025.100854 (PMC12329596; doi:10.1016/j.xops.2025.100854)
Supplement: Table S6 [file mmc7.pdf]

.metric	.estimator	.estimate	dataset	model
accuracy	multiclass	0.88409311	train	multinom
kap	multiclass	0.55433942	train	multinom
sens	macro	0.63795803	train	multinom
spec	macro	0.83962765	train	multinom
ppv	macro	0.75416624	train	multinom
npv	macro	0.90844521	train	multinom
mcc	multiclass	0.56740617	train	multinom
j_index	macro	0.47758568	train	multinom
bal_accura	macro	0.73879284	train	multinom
detection_	macro	0.33333333	train	multinom
precision	macro	0.75416624	train	multinom
recall	macro	0.63795803	train	multinom
f_meas	macro	0.68319151	train	multinom
roc_auc	hand_till	0.88098674	train	multinom
accuracy	multiclass	0.88124091	test	multinom
kap	multiclass	0.53634495	test	multinom
sens	macro	0.63599632	test	multinom
spec	macro	0.83655680	test	multinom
ppv	macro	0.72777148	test	multinom
npv	macro	0.89604520	test	multinom
mcc	multiclass	0.54662996	test	multinom
j_index	macro	0.47255312	test	multinom
bal_accura	macro	0.73627656	test	multinom
detection_	macro	0.33333333	test	multinom
precision	macro	0.72777148	test	multinom
recall	macro	0.63599632	test	multinom
f_meas	macro	0.67260056	test	multinom
roc_auc	hand_till	0.87124610	test	multinom
