# Supplementary material for: Integration of Immunometabolic Composite Indices and Machine Learning for Diabetic Retinopathy Risk Stratification: Insights from NHANES 2011 – 2020
Source: Ophthalmol Sci. 2025 Jun 16;5(6):100854. doi: 10.1016/j.xops.2025.100854 (PMC12329596; doi:10.1016/j.xops.2025.100854)
Supplement: Table S7 [file mmc8.pdf]

.metric	.estimator	.estimate	dataset	model
accuracy	multiclass	0.90995796	train	rf
kap	multiclass	0.64315926	train	rf
sens	macro	0.66872120	train	rf
spec	macro	0.85810963	train	rf
ppv	macro	0.88543459	train	rf
npv	macro	0.95528093	train	rf
mcc	multiclass	0.66568052	train	rf
j_index	macro	0.52683083	train	rf
bal_accura	macro	0.76341541	train	rf
detection_	macro	0.33333333	train	rf
precision	macro	0.88543459	train	rf
recall	macro	0.66872120	train	rf
f_meas	macro	0.74220530	train	rf
roc_auc	hand_till	0.95168809	train	rf
accuracy	multiclass	0.90208434	test	rf
kap	multiclass	0.59355383	test	rf
sens	macro	0.65197227	test	rf
spec	macro	0.83705841	test	rf
ppv	macro	0.88321719	test	rf
npv	macro	0.93618038	test	rf
mcc	multiclass	0.61920648	test	rf
j_index	macro	0.48903068	test	rf
bal_accura	macro	0.74451534	test	rf
detection_	macro	0.33333333	test	rf
precision	macro	0.88321719	test	rf
recall	macro	0.65197227	test	rf
f_meas	macro	0.72949688	test	rf
roc_auc	hand_till	0.88905894	test	rf
