# Supplementary material for: Integration of Immunometabolic Composite Indices and Machine Learning for Diabetic Retinopathy Risk Stratification: Insights from NHANES 2011 – 2020
Source: Ophthalmol Sci. 2025 Jun 16;5(6):100854. doi: 10.1016/j.xops.2025.100854 (PMC12329596; doi:10.1016/j.xops.2025.100854)
Supplement: Table S8 [file mmc9.pdf]

.metric	.estimator	.estimate	dataset	model
accuracy	multiclass	0.89589395	train	psvm
kap	multiclass	0.59305125	train	psvm
sens	macro	0.65306545	train	psvm
spec	macro	0.84742728	train	psvm
ppv	macro	0.81255928	train	psvm
npv	macro	0.93011344	train	psvm
mcc	multiclass	0.61049822	train	psvm
j_index	macro	0.50049274	train	psvm
bal_accura	macro	0.75024637	train	psvm
detection_	macro	0.33333333	train	psvm
precision	macro	0.81255928	train	psvm
recall	macro	0.65306545	train	psvm
f_meas	macro	0.71241216	train	psvm
roc_auc	hand_till	0.88663368	train	psvm
accuracy	multiclass	0.89481337	test	psvm
kap	multiclass	0.58539729	test	psvm
sens	macro	0.65383606	test	psvm
spec	macro	0.84932976	test	psvm
ppv	macro	0.77782527	test	psvm
npv	macro	0.91860177	test	psvm
mcc	multiclass	0.59805118	test	psvm
j_index	macro	0.50316582	test	psvm
bal_accura	macro	0.75158291	test	psvm
detection_	macro	0.33333333	test	psvm
precision	macro	0.77782527	test	psvm
recall	macro	0.65383606	test	psvm
f_meas	macro	0.70268784	test	psvm
roc_auc	hand_till	0.87545513	test	psvm
