# Supplementary material for: Integration of Immunometabolic Composite Indices and Machine Learning for Diabetic Retinopathy Risk Stratification: Insights from NHANES 2011 – 2020
Source: Ophthalmol Sci. 2025 Jun 16;5(6):100854. doi: 10.1016/j.xops.2025.100854 (PMC12329596; doi:10.1016/j.xops.2025.100854)
Supplement: Figure S11 [file mmc11.pdf]

FigureS11

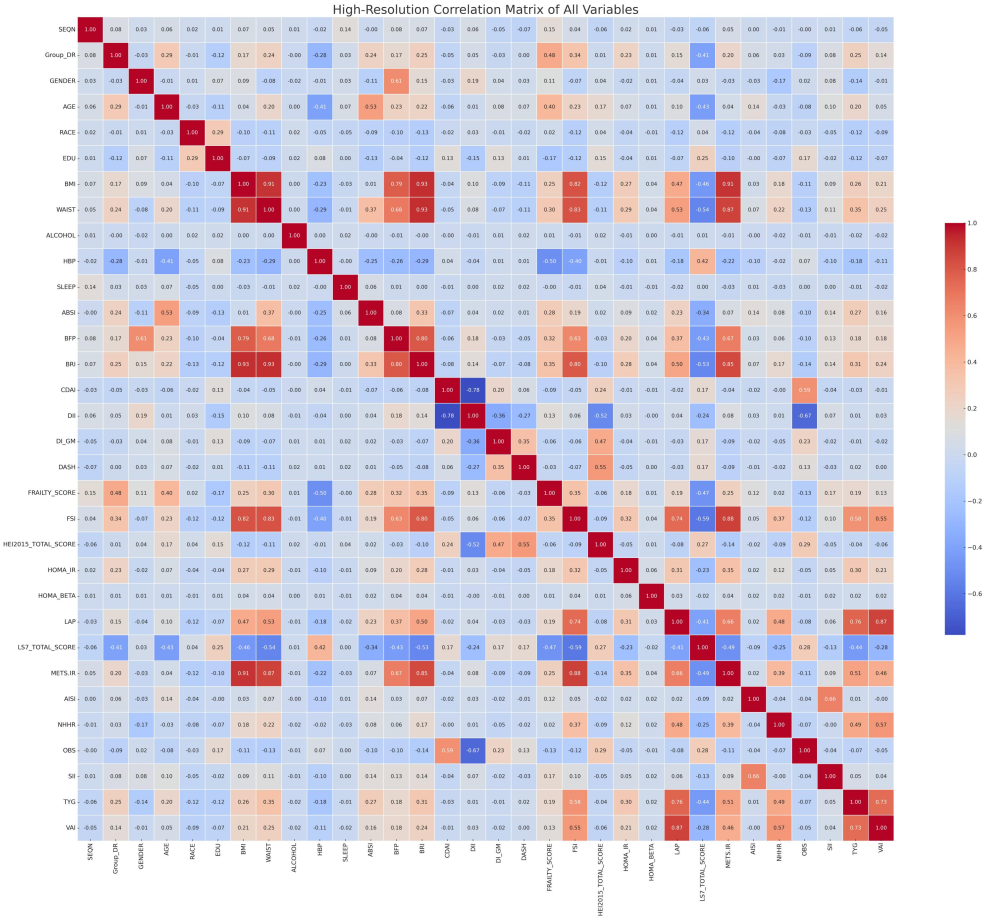

Figure S11. Correlation heatmap of all variables used in the study. Pearson correlation coefficients among all included immunometabolic indices and clinical-demographic variables are shown. The intensity and color of each square indicate the magnitude and direction (positive correlations in red, negative correlations in blue) of the correlation coefficient, as depicted by the color gradient scale.
