# Supplementary material for: Integration of Immunometabolic Composite Indices and Machine Learning for Diabetic Retinopathy Risk Stratification: Insights from NHANES 2011 – 2020
Source: Ophthalmol Sci. 2025 Jun 16;5(6):100854. doi: 10.1016/j.xops.2025.100854 (PMC12329596; doi:10.1016/j.xops.2025.100854)
Supplement: Figure S12 [file mmc12.pdf]

FigureS12

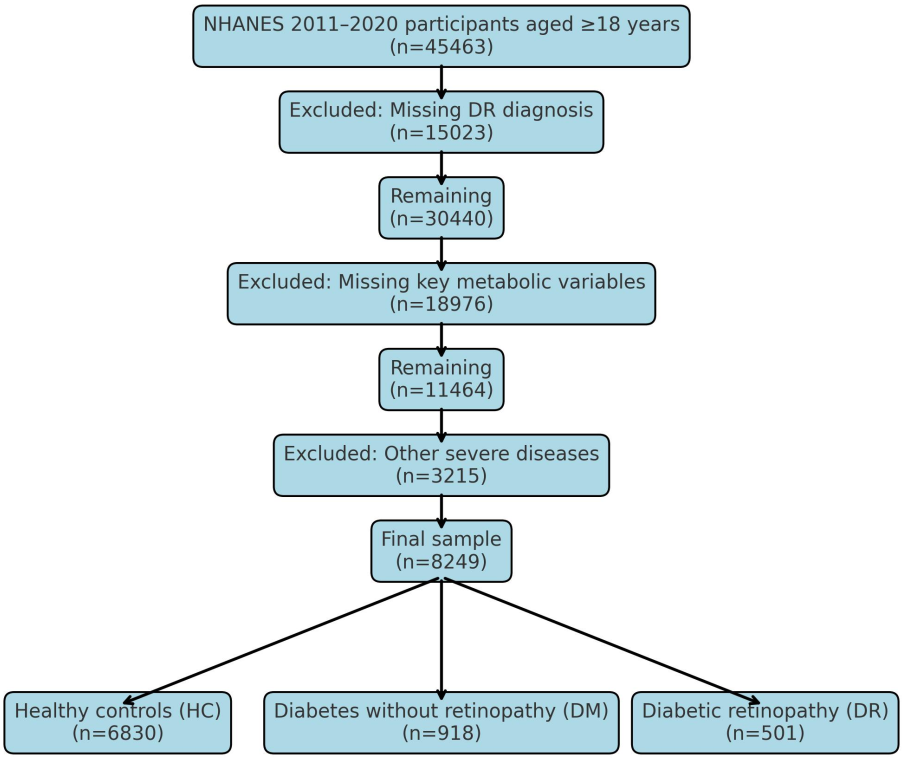

Figure S12. Flowchart depicting the selection of study participants from NHANES 2011-2020.

Participants aged ≥18 years from NHANES datasets underwent sequential exclusion criteria: missing diabetic retinopathy (DR) diagnosis, incomplete key metabolic variables, and presence of other severe diseases. The final analytic sample (n=8249) was categorized into three groups: Healthy controls (HC, n=6830), Diabetes without retinopathy (DM, n=918), and Diabetic retinopathy (DR, n=501).
