# Supplementary material for: Integration of Immunometabolic Composite Indices and Machine Learning for Diabetic Retinopathy Risk Stratification: Insights from NHANES 2011 – 2020
Source: Ophthalmol Sci. 2025 Jun 16;5(6):100854. doi: 10.1016/j.xops.2025.100854 (PMC12329596; doi:10.1016/j.xops.2025.100854)
Supplement: Figure S2 [file mmc13.pdf]

Figure S2

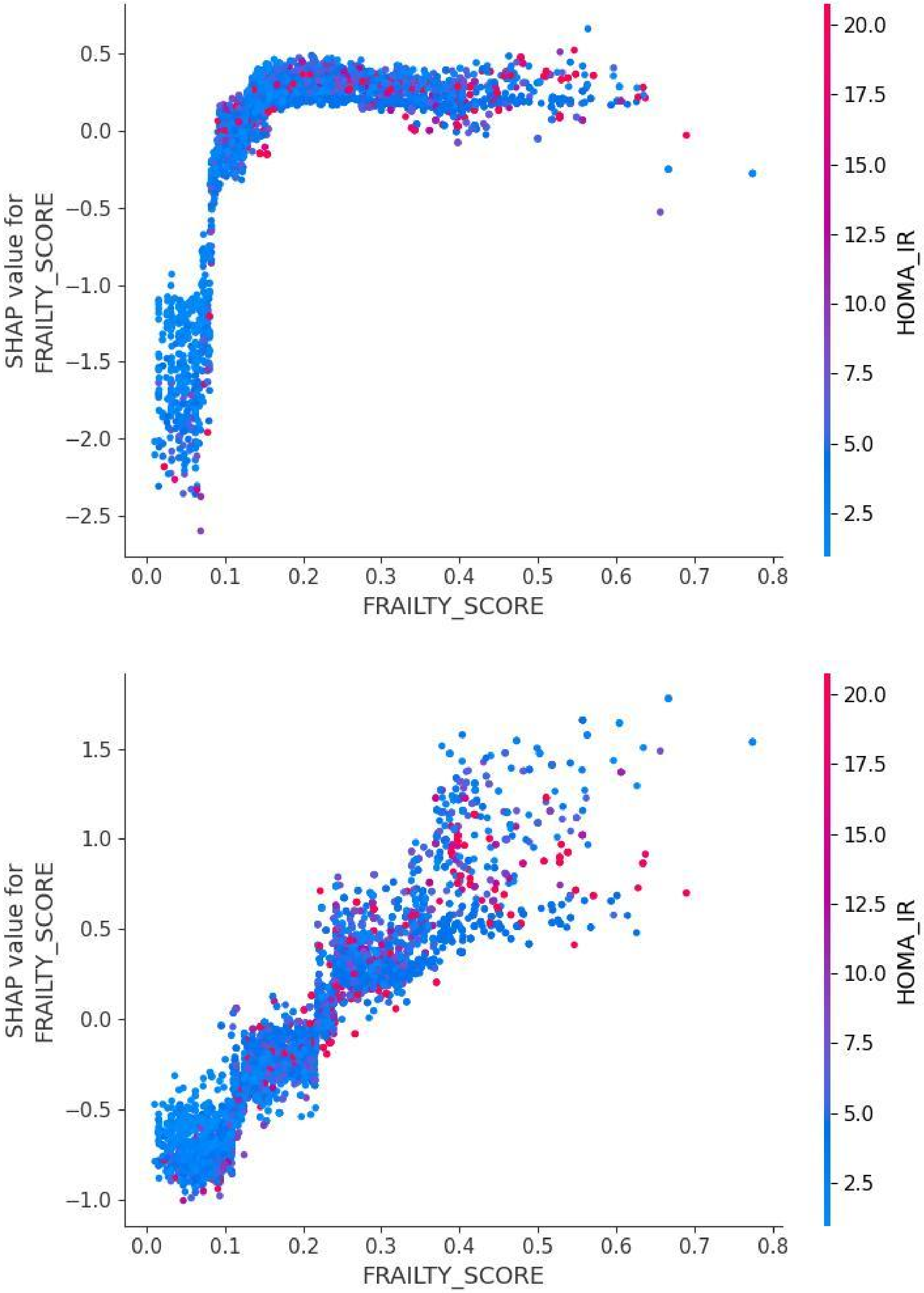
SHAP value for FRAILTY_SCORE
0.5
0.0
−0.5
−1.0
−1.5
−2.0
−2.5
0.0 0.1 0.2 0.3 0.4 0.5 0.6 0.7 0.8
FRAILTY_SCORE
HOMA_IR
20.0
17.5
15.0
12.5
10.0
7.5
5.0
2.5
SHAP value for FRAILTY_SCORE
1.5
1.0
0.5
0.0
−0.5
−1.0
0.0 0.1 0.2 0.3 0.4 0.5 0.6 0.7 0.8
FRAILTY_SCORE
HOMA_IR
20.0
17.5
15.0
12.5
10.0
7.5
5.0
2.5

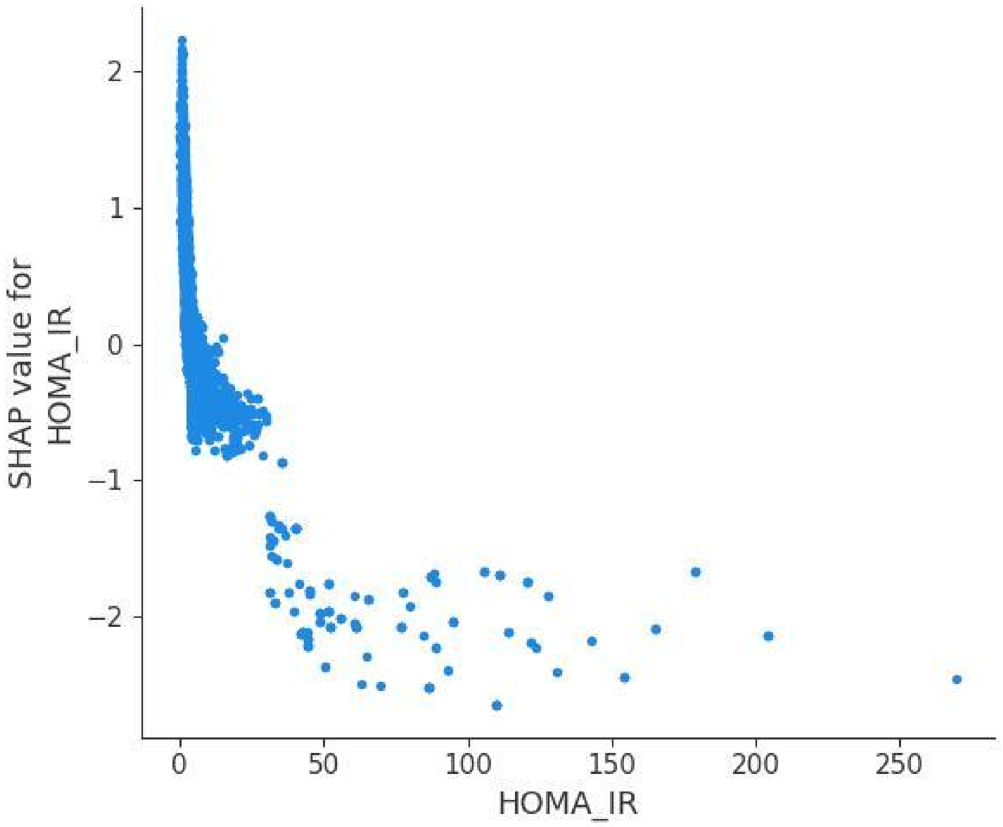

SHAP value for HOMA_IR
HOMA_IR
2
1
0
−1
−2
0
50
100
150
200
250

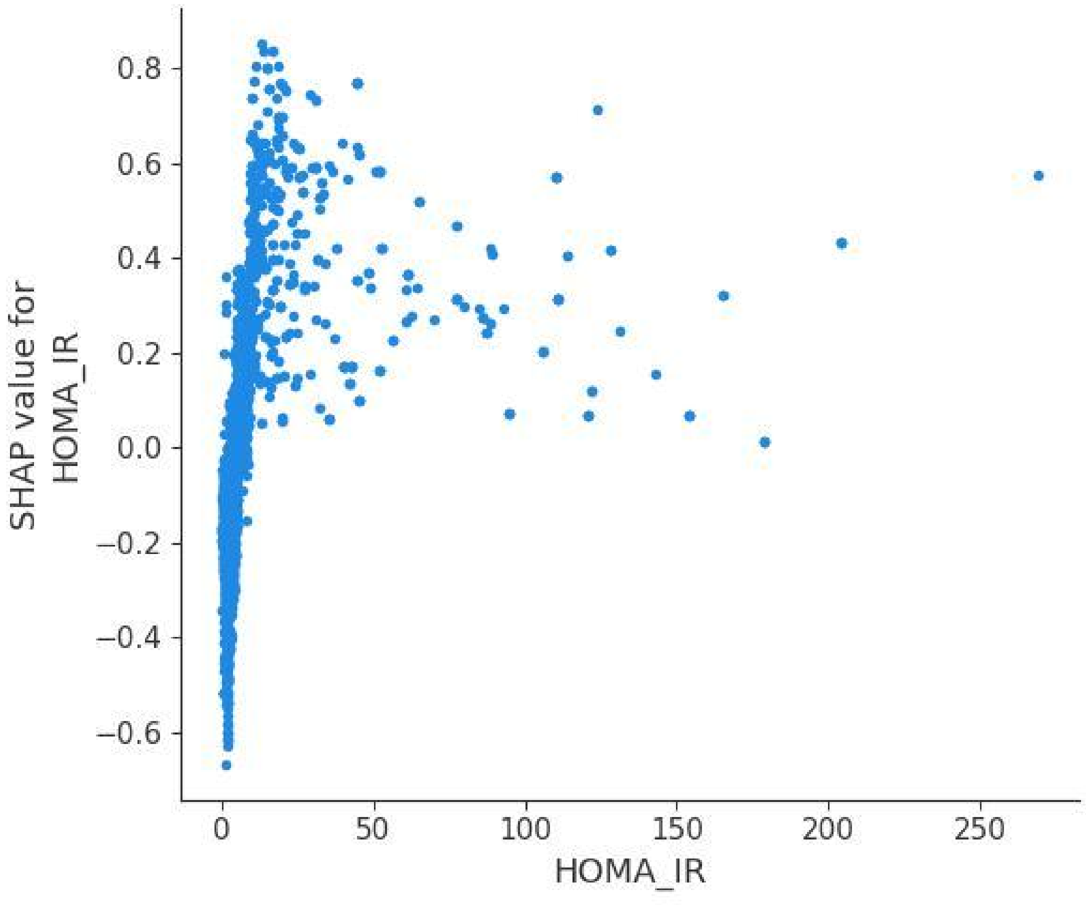

SHAP value for HOMA_IR
HOMA_IR
0.8
0.6
0.4
0.2
0.0
−0.2
−0.4
−0.6
0
50
100
150
200
250

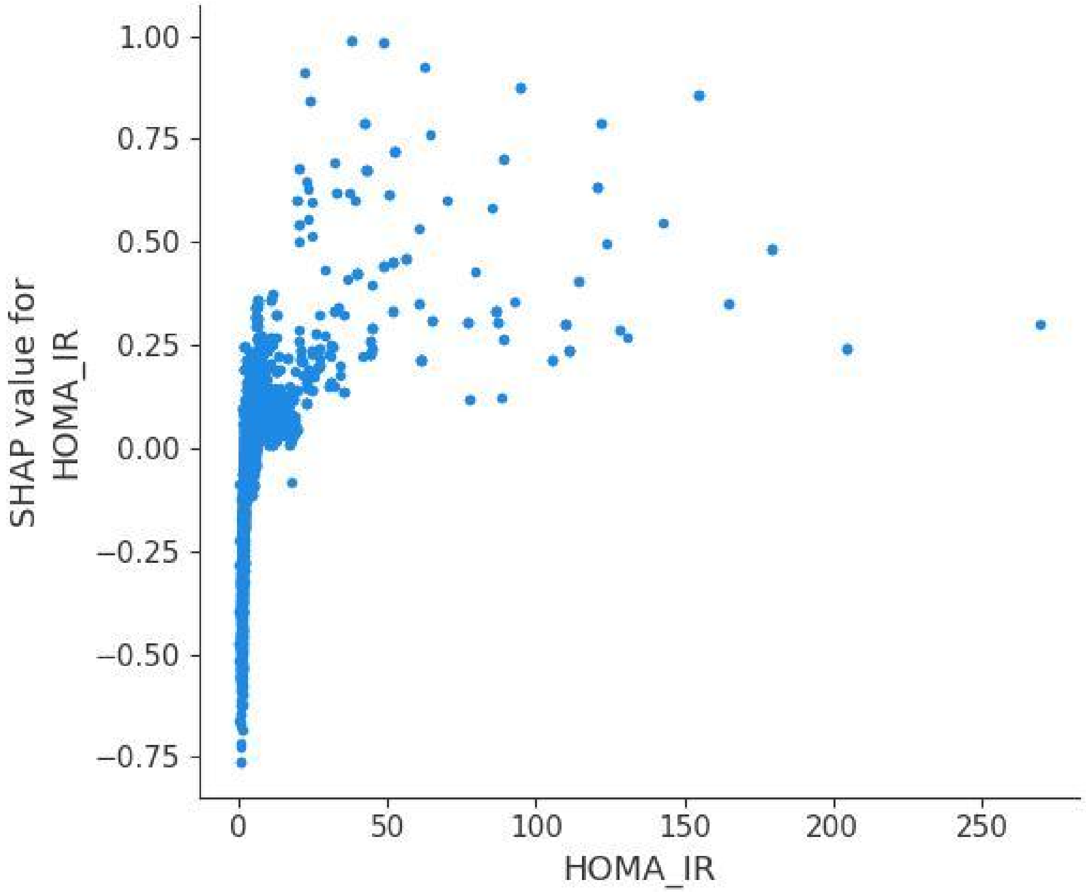
SHAP value for HOMA_IR
1.00
0.75
0.50
0.25
0.00
−0.25
−0.50
−0.75
0
50
100
150
200
250
HOMA_IR

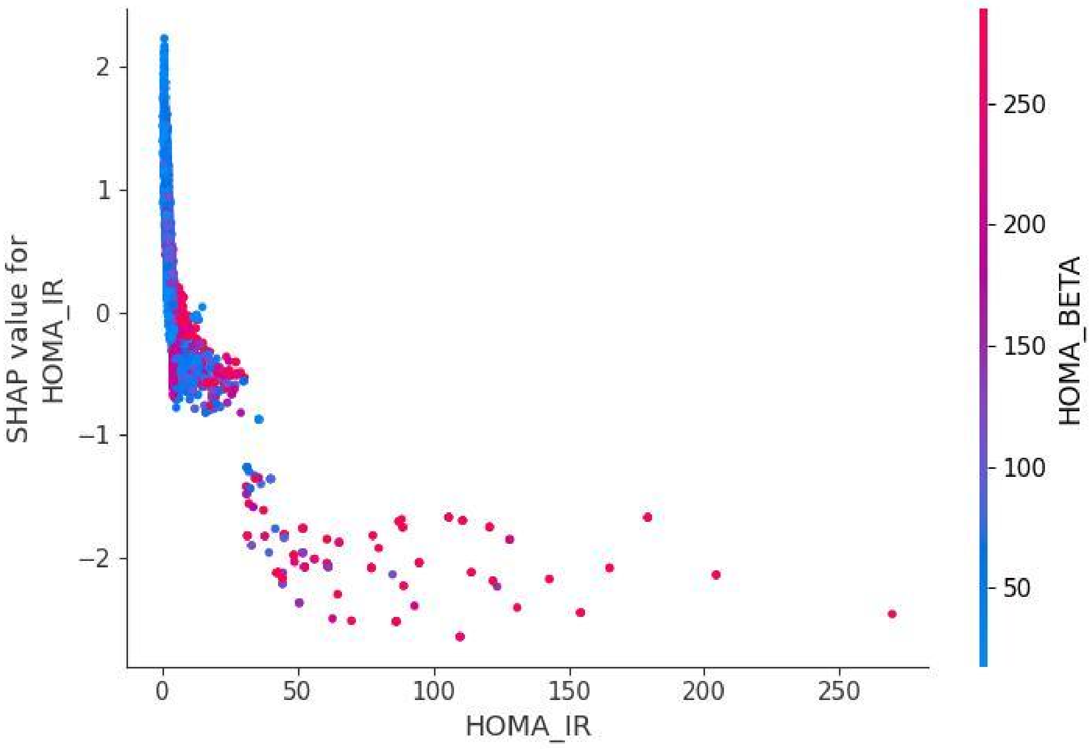
SHAP value for HOMA_IR
2
1
0
−1
−2
0
50
100
150
200
250
HOMA_IR
HOMA_BETA
250
200
150
100
50

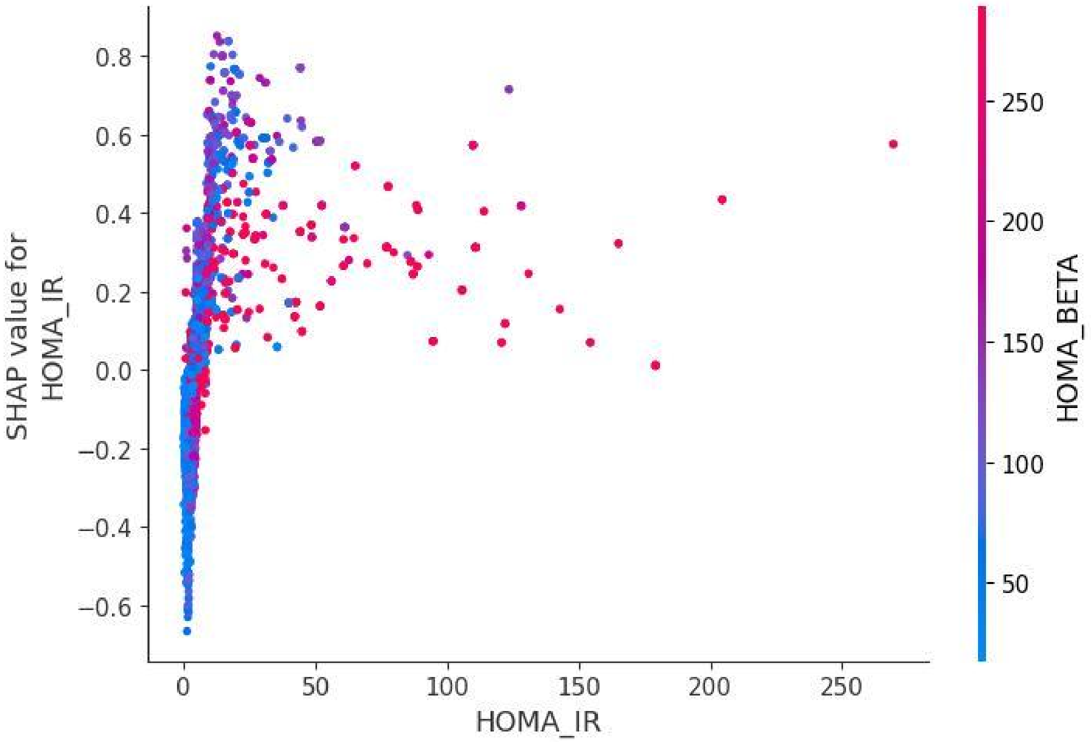

0.8
0.6
0.4
0.2
0.0
−0.2
−0.4
−0.6
SHAP value for
HOMA_IR
0
50
100
150
200
250
HOMA_IR
250
200
150
100
50
HOMA_BETA

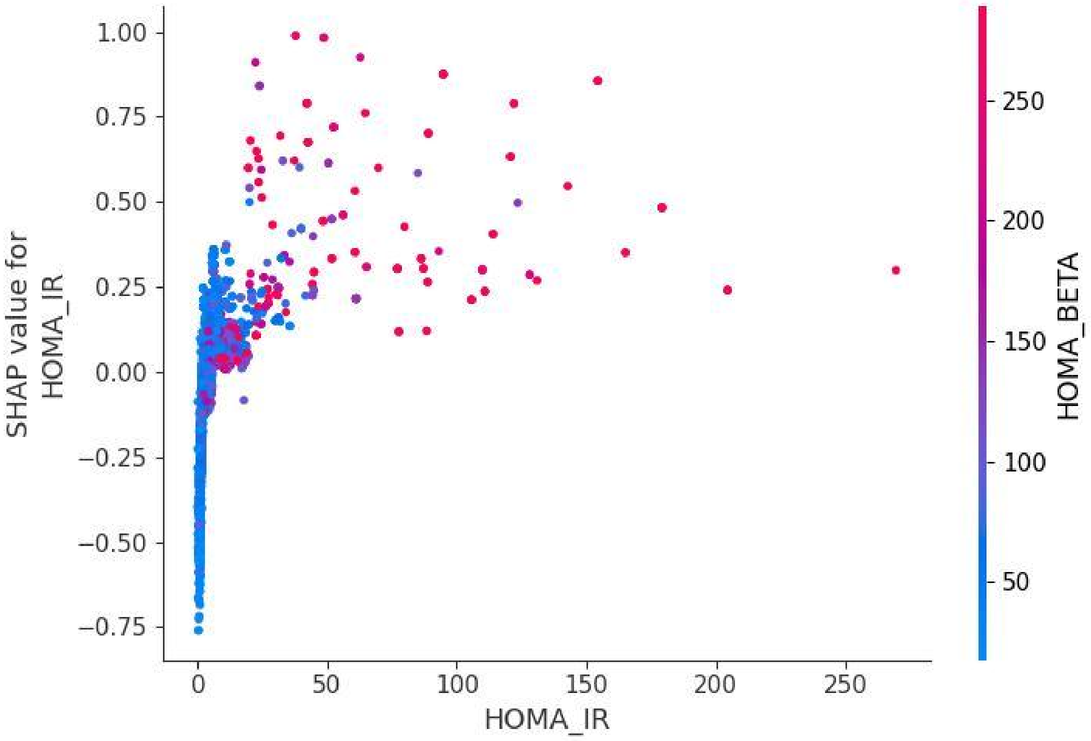

1.00
0.75
0.50
0.25
0.00
−0.25
−0.50
−0.75
SHAP value for
HOMA_IR
0
50
100
150
200
250
HOMA_IR
250
200
150
100
50
HOMA_BETA

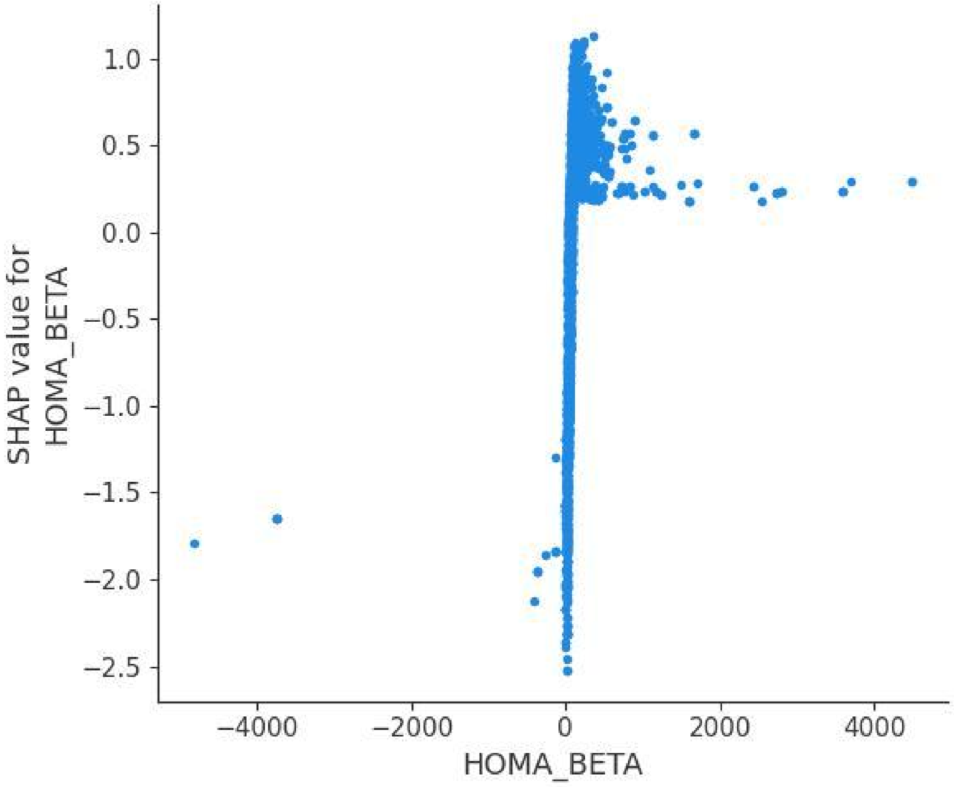

SHAP value for HOMA_BETA
HOMA_BETA
1.0
0.5
0.0
−0.5
−1.0
−1.5
−2.0
−2.5
−4000
−2000
0
2000
4000

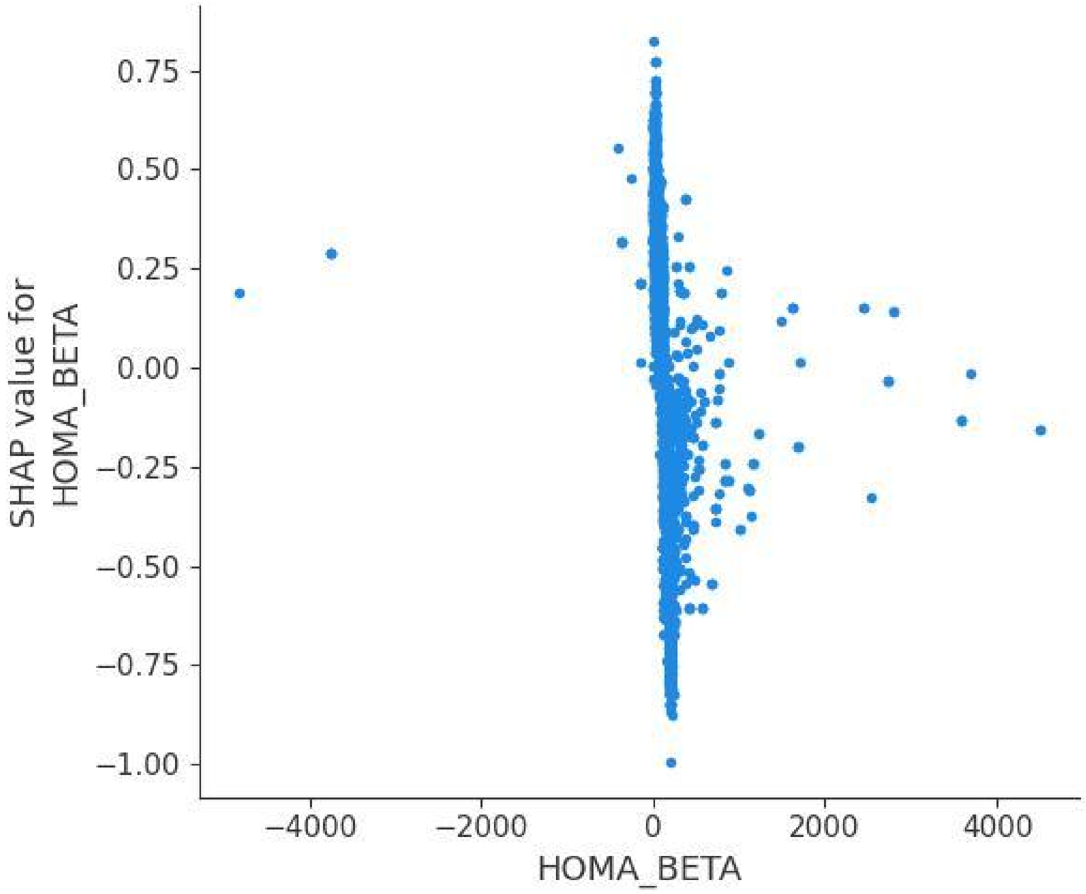

SHAP value for HOMA_BETA
HOMA_BETA
0.75
0.50
0.25
0.00
−0.25
−0.50
−0.75
−1.00
−4000
−2000
0
2000
4000

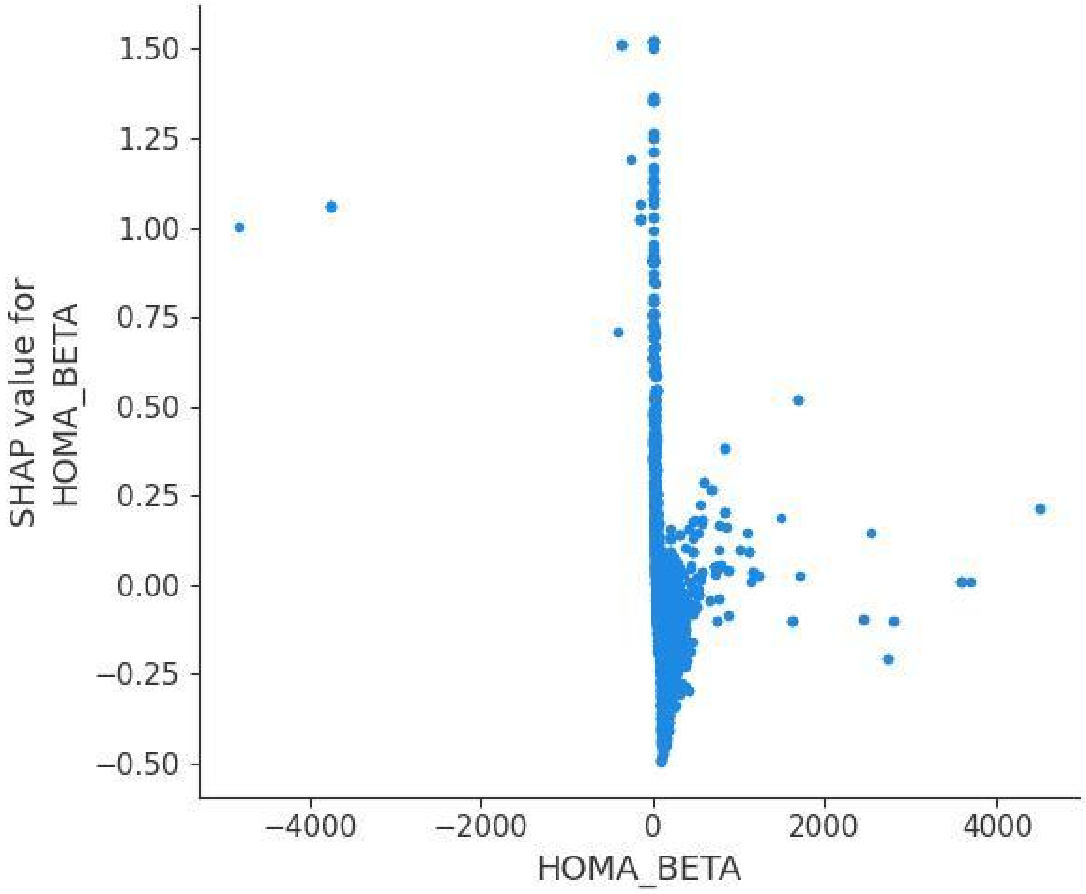
SHAP value for HOMA_BETA
HOMA_BETA

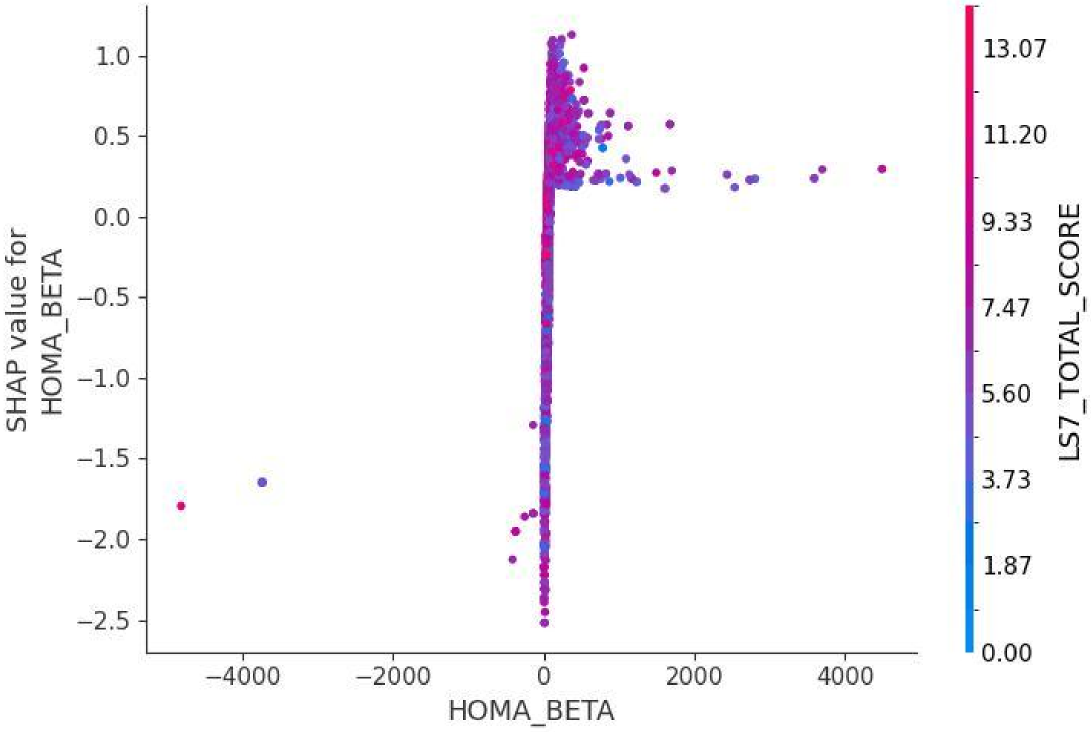
SHAP value for HOMA_BETA
HOMA_BETA
LS7_TOTAL_SCORE

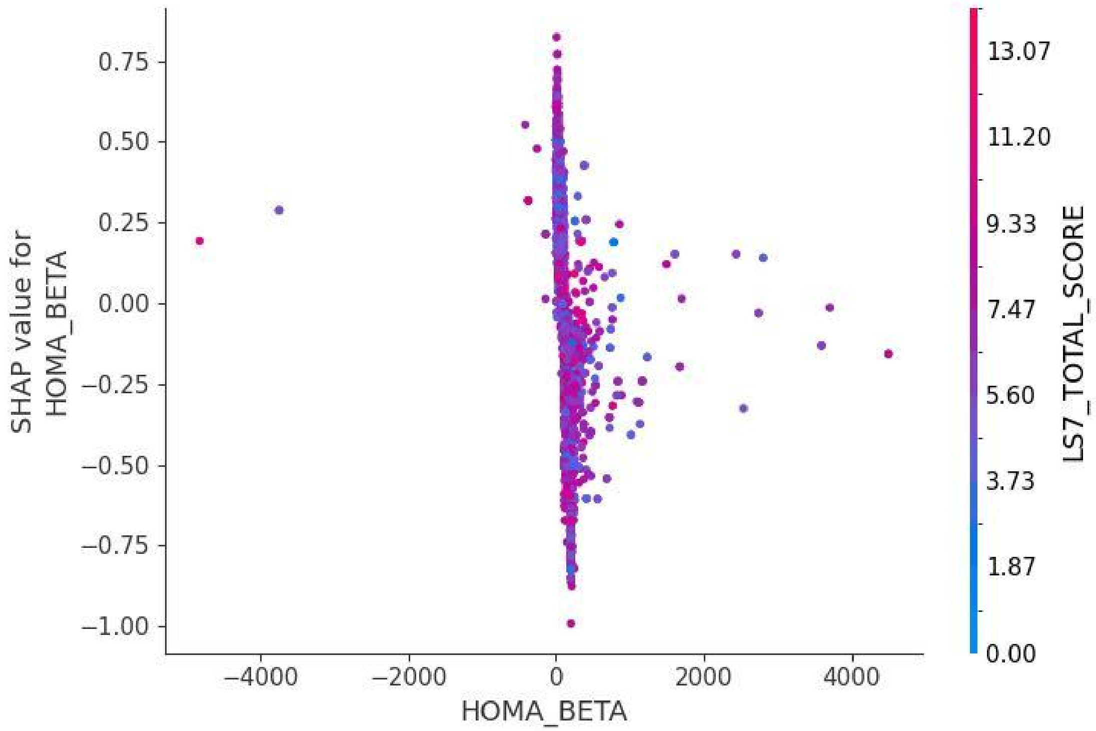
SHAP value for HOMA_BETA
0.75
0.50
0.25
0.00
−0.25
−0.50
−0.75
−1.00
−4000
−2000
0
2000
4000
HOMA_BETA
13.07
11.20
9.33
7.47
5.60
3.73
1.87
0.00
LS7_TOTAL_SCORE

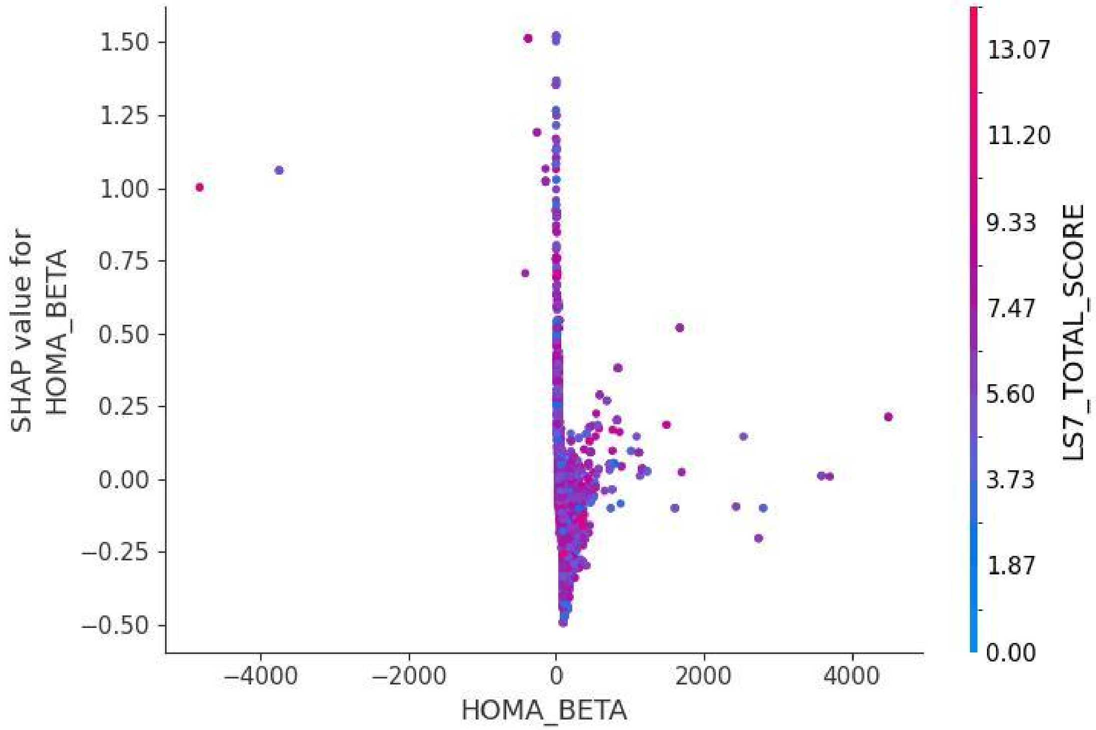
SHAP value for HOMA_BETA
1.50
1.25
1.00
0.75
0.50
0.25
0.00
−0.25
−0.50
−4000
−2000
0
2000
4000
HOMA_BETA
13.07
11.20
9.33
7.47
5.60
3.73
1.87
0.00
LS7_TOTAL_SCORE

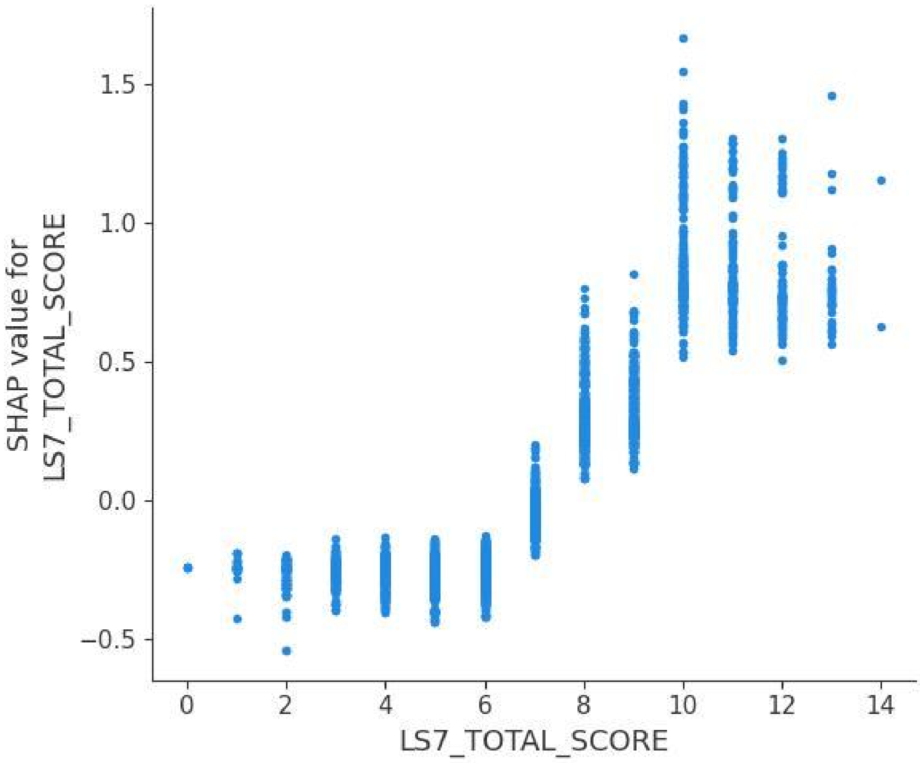

1.5
1.0
0.5
0.0
−0.5
SHAP value for LS7_TOTAL_SCORE
0
2
4
6
8
10
12
14
LS7_TOTAL_SCORE

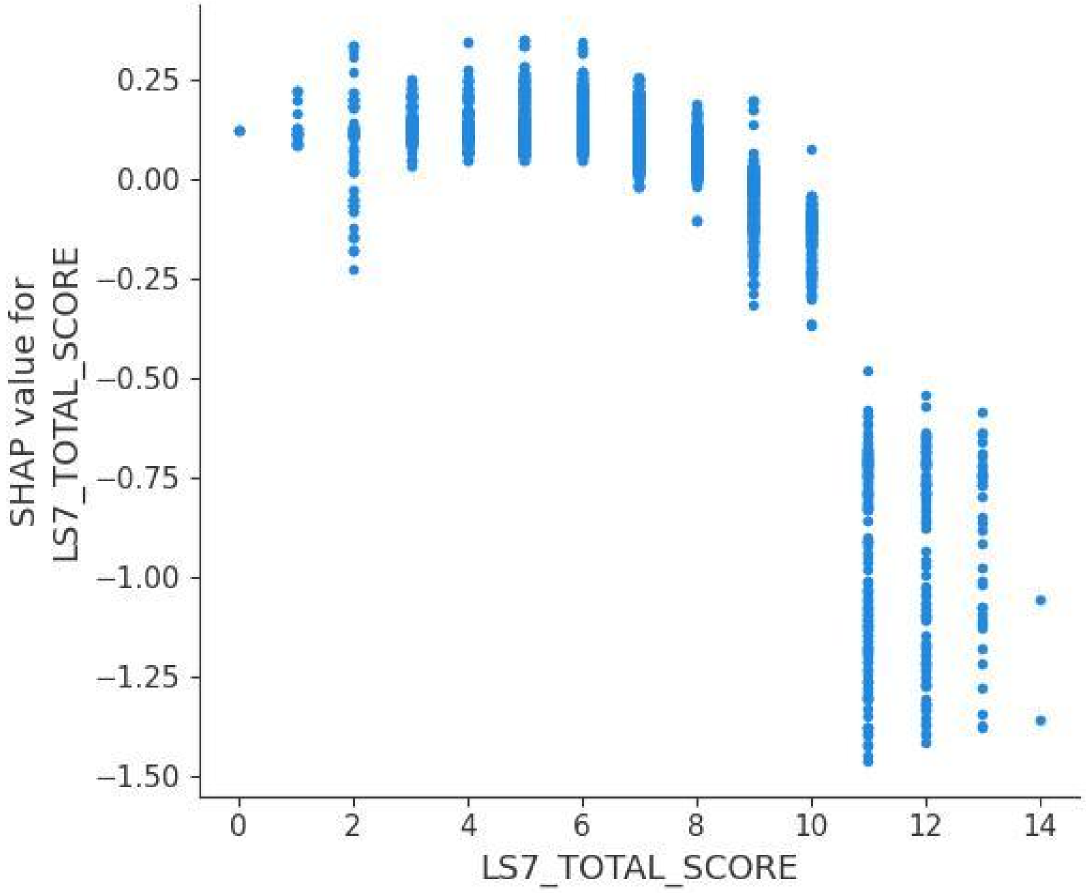

0.25
0.00
−0.25
−0.50
−0.75
−1.00
−1.25
−1.50
SHAP value for LS7_TOTAL_SCORE
0
2
4
6
8
10
12
14
LS7_TOTAL_SCORE

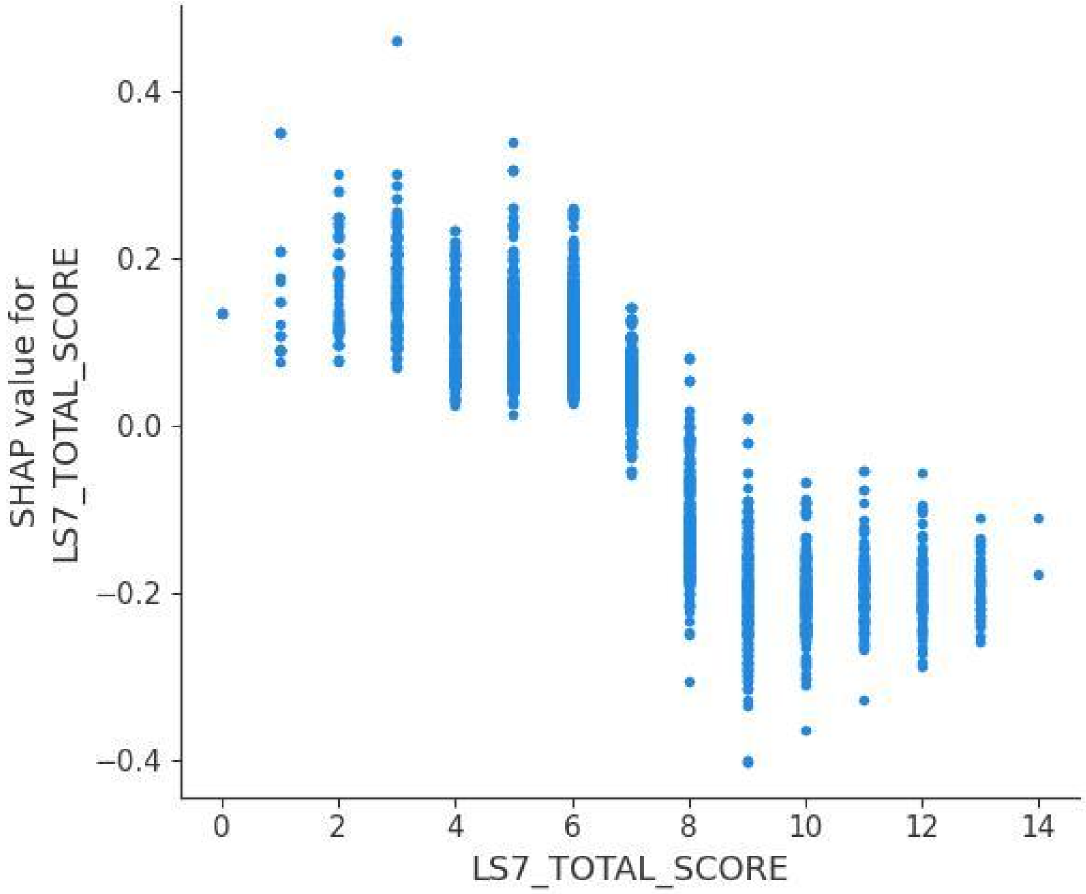

SHAP value for
LS7_TOTAL_SCORE
0.4
0.2
0.0
−0.2
−0.4
0
2
4
6
8
10
12
14
LS7_TOTAL_SCORE

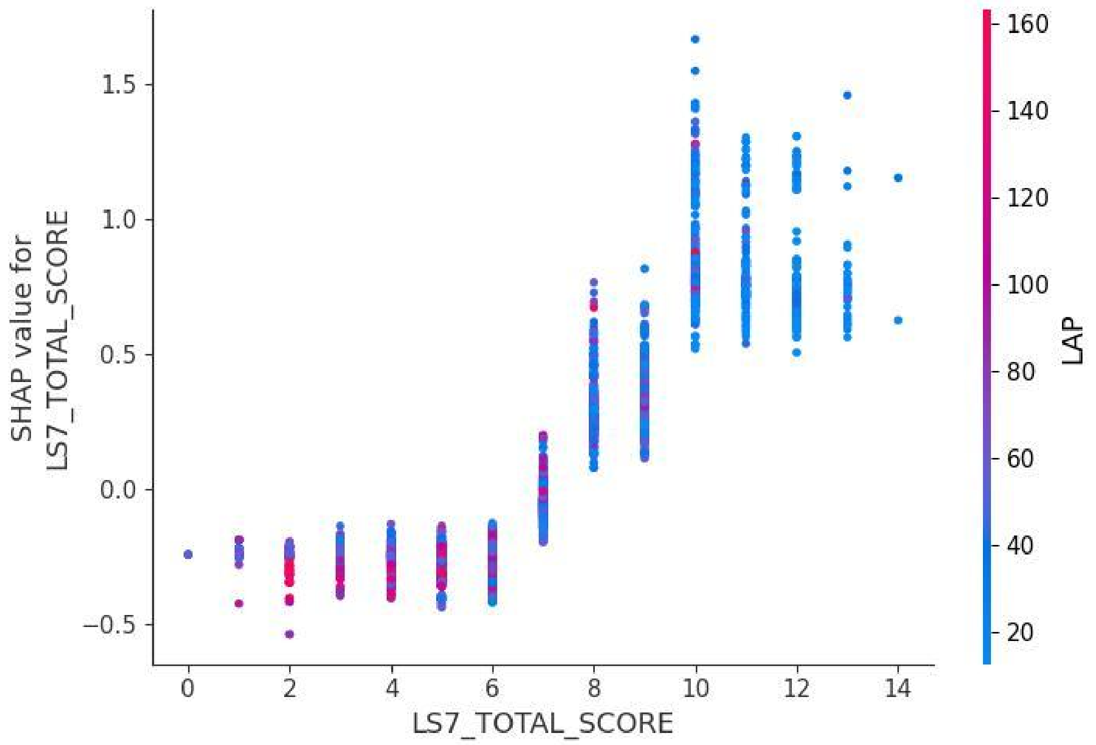

SHAP value for
LS7_TOTAL_SCORE
1.5
1.0
0.5
0.0
−0.5
0
2
4
6
8
10
12
14
LS7_TOTAL_SCORE
160
140
120
100
80
60
40
20
LAP

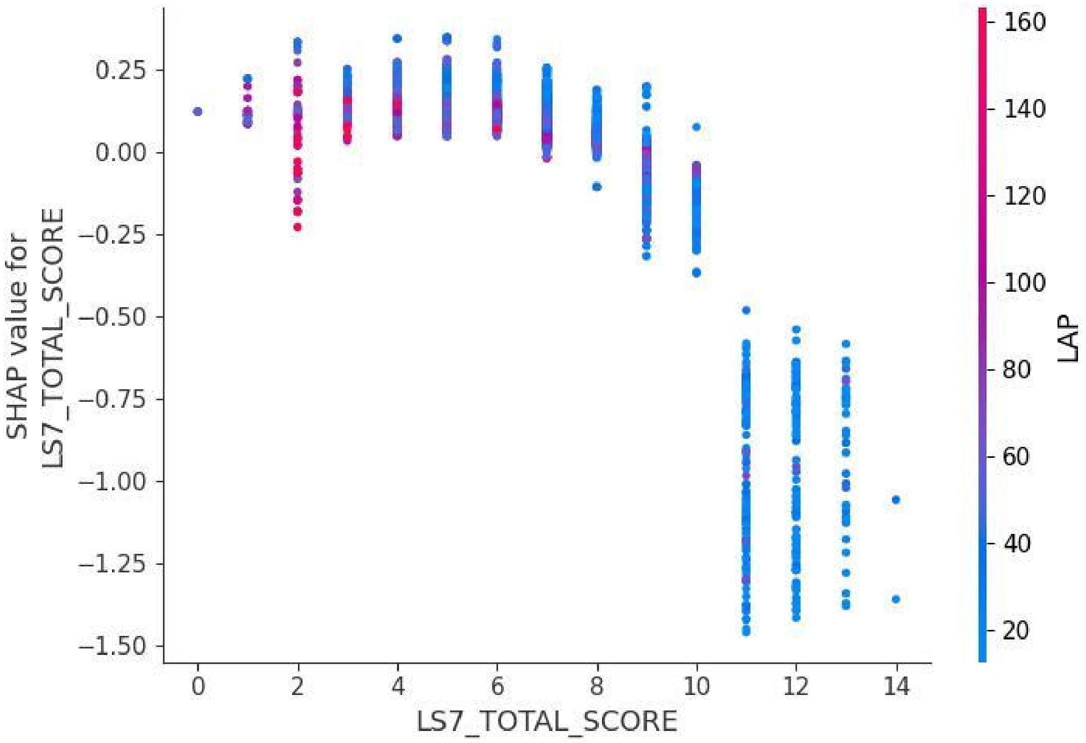
SHAP value for LS7_TOTAL_SCORE
LS7_TOTAL_SCORE
LAP
0.25
0.00
−0.25
−0.50
−0.75
−1.00
−1.25
−1.50
0
2
4
6
8
10
12
14
160
140
120
100
80
60
40
20

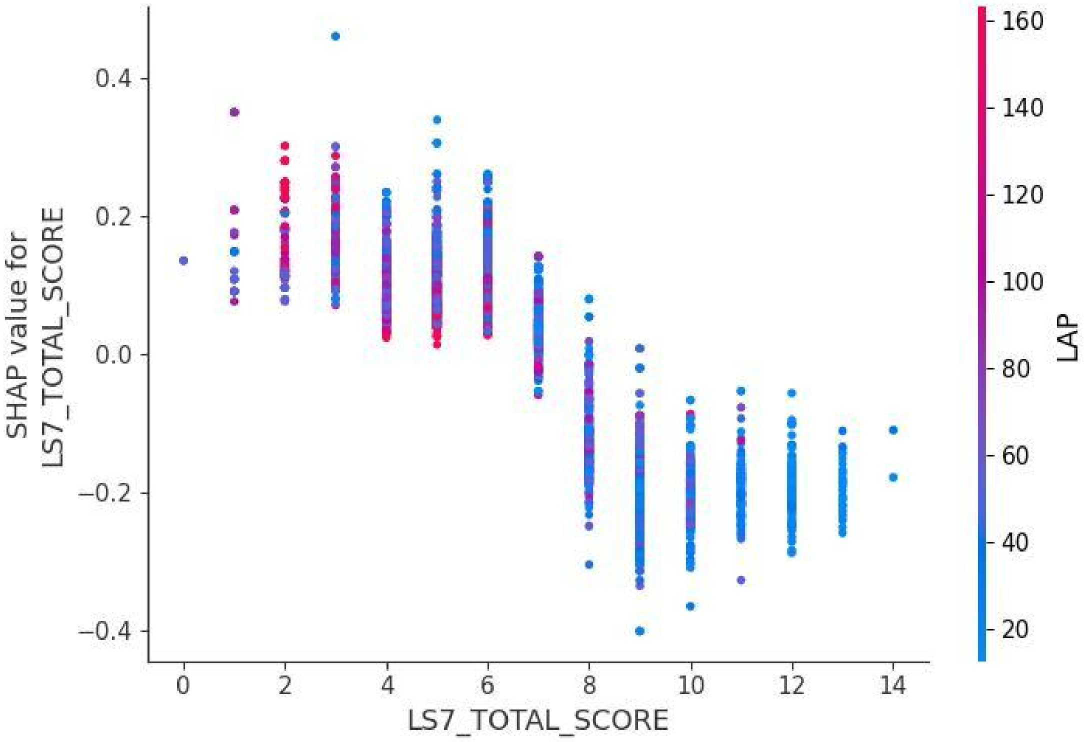
SHAP value for LS7_TOTAL_SCORE
LS7_TOTAL_SCORE
LAP
0.4
0.2
0.0
−0.2
−0.4
0
2
4
6
8
10
12
14
160
140
120
100
80
60
40
20

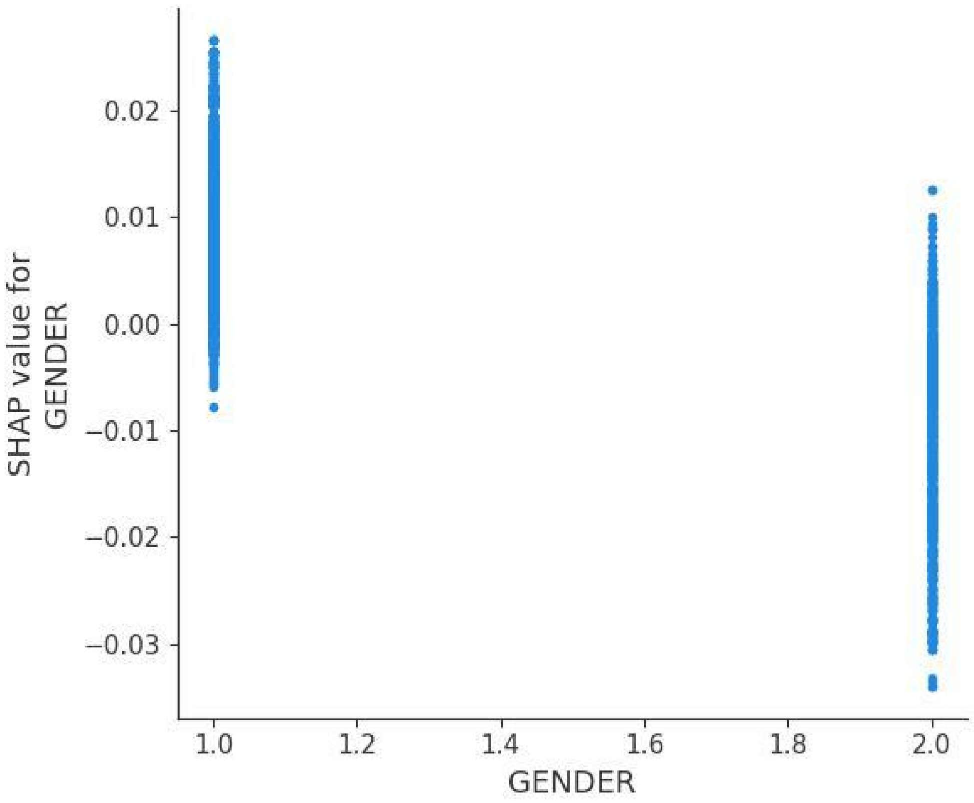
SHAP value for GENDER
0.02
0.01
0.00
−0.01
−0.02
−0.03
1.0
1.2
1.4
1.6
1.8
2.0
GENDER

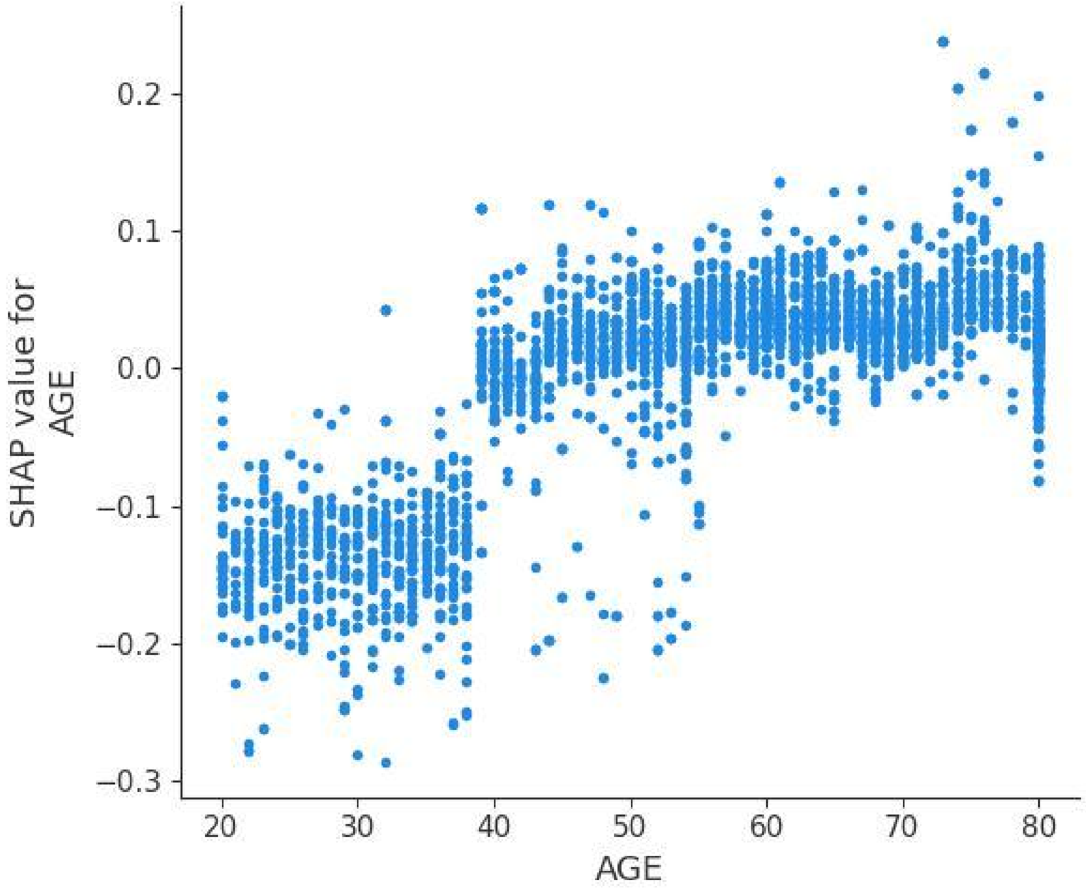
SHAP value for AGE
0.2
0.1
0.0
−0.1
−0.2
−0.3
20
30
40
50
60
70
80
AGE

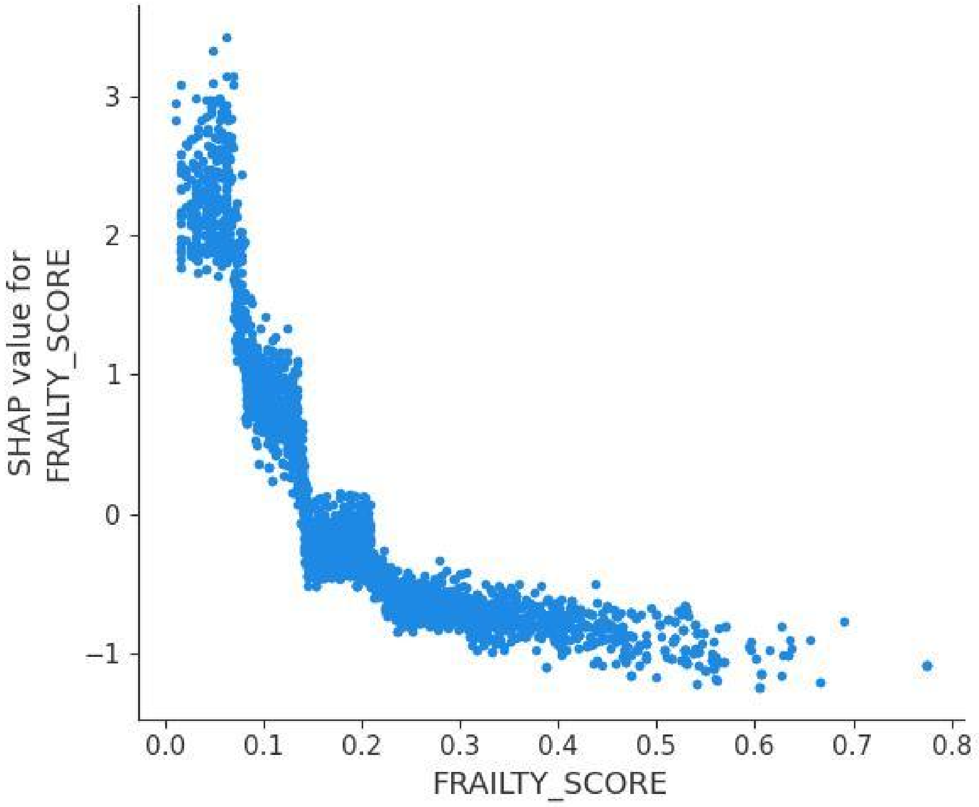

SHAP value for FRAILTY_SCORE
FRAILTY_SCORE
3
2
1
0
−1
0.0
0.1
0.2
0.3
0.4
0.5
0.6
0.7
0.8

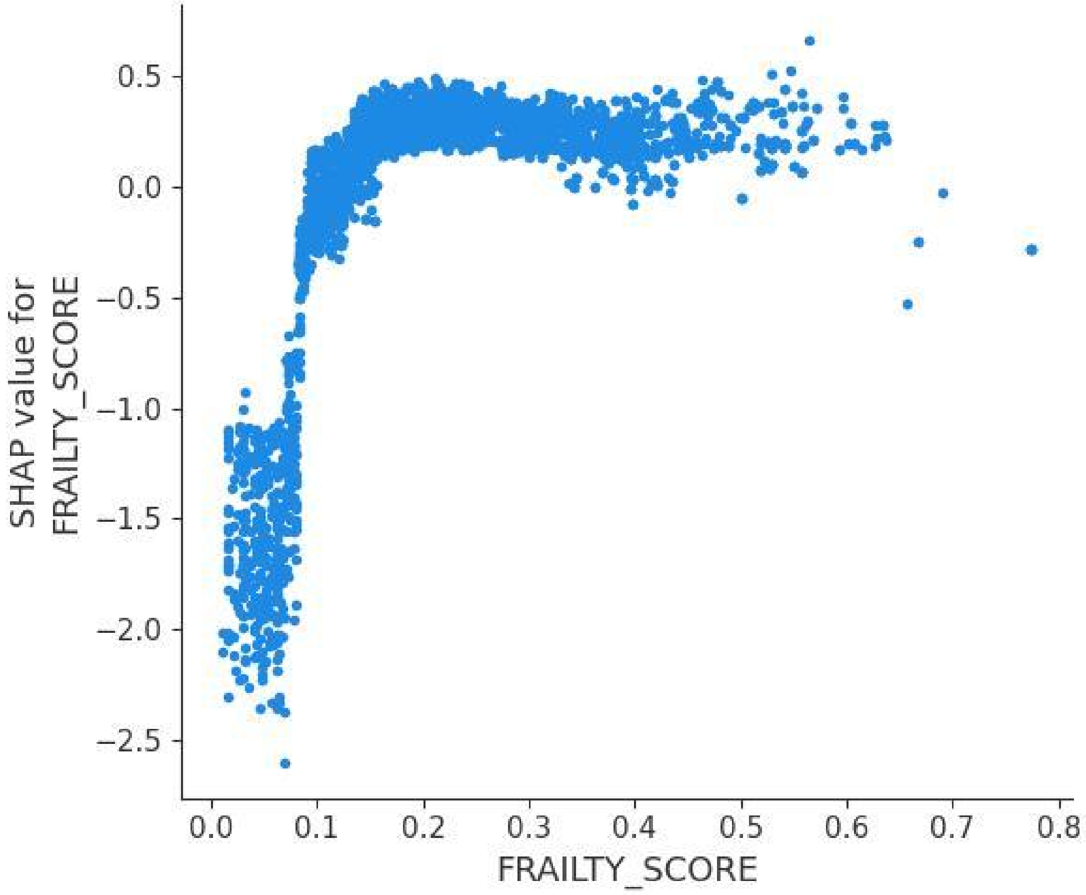

SHAP value for FRAILTY_SCORE
FRAILTY_SCORE
0.5
0.0
−0.5
−1.0
−1.5
−2.0
−2.5
0.0
0.1
0.2
0.3
0.4
0.5
0.6
0.7
0.8

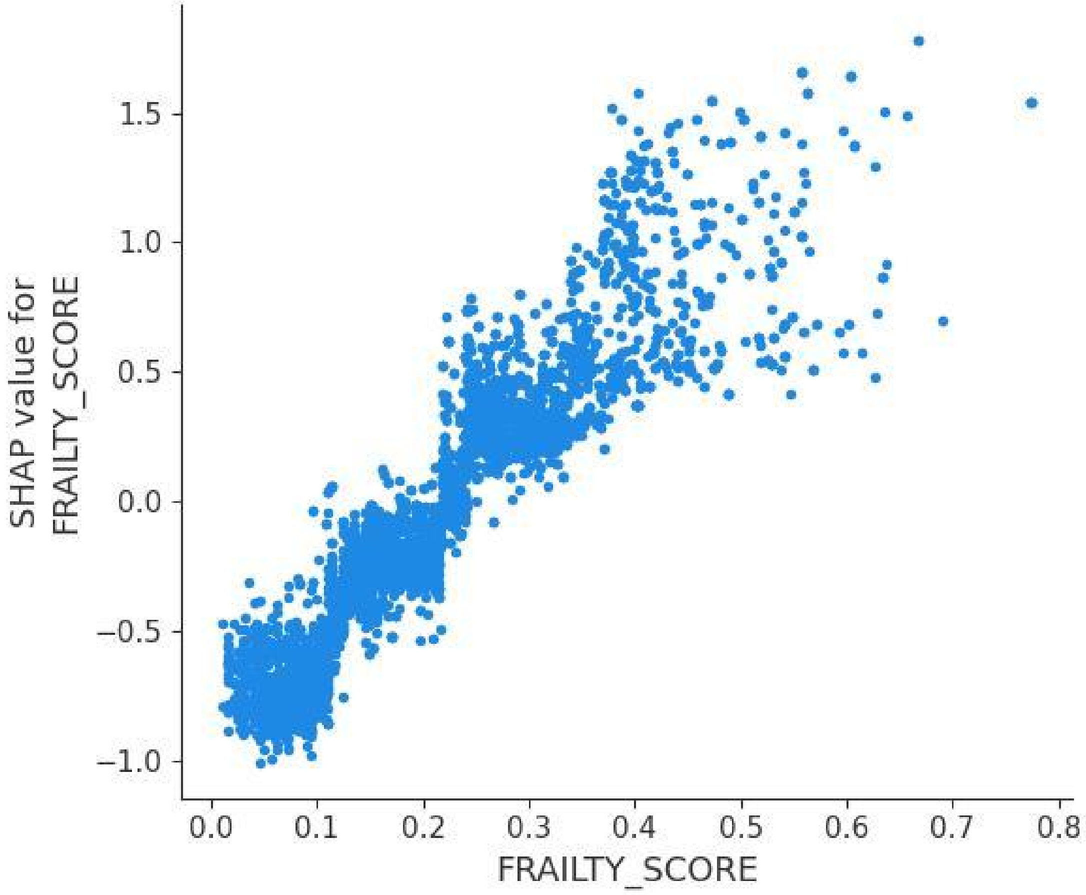

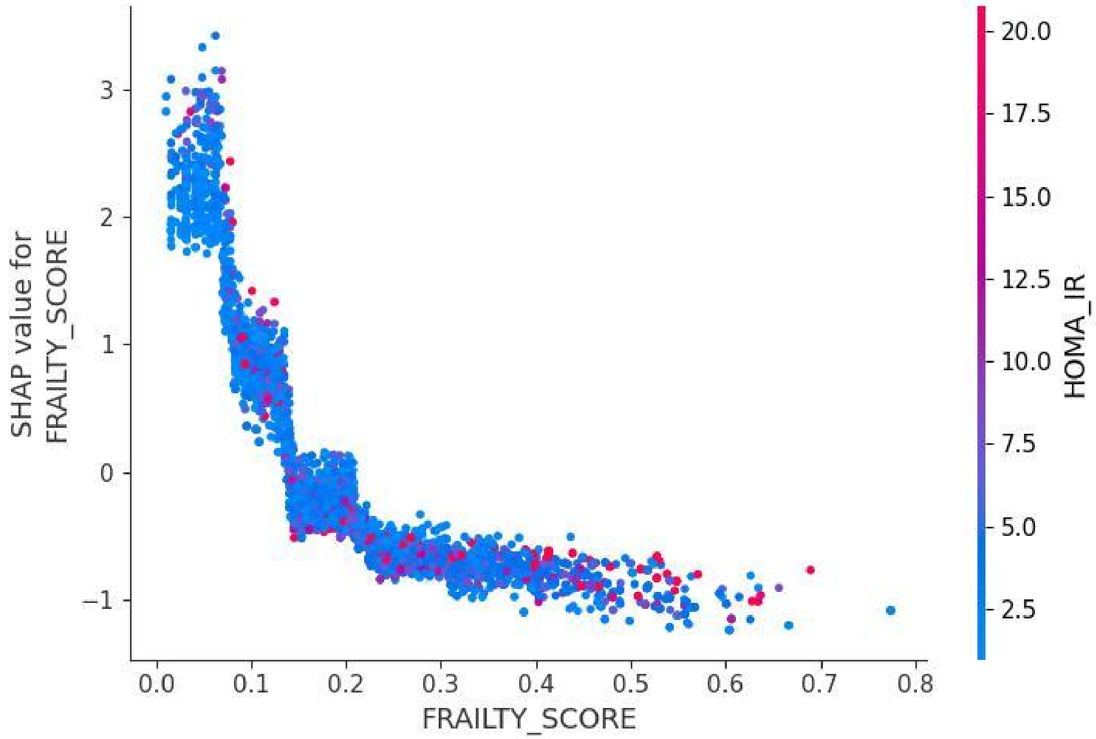

Figure S2. SHAP (SHapley Additive exPlanations) value plots demonstrating the impact of different clinical indices on diabetic retinopathy risk stratification.
Each subplot illustrates the contribution (SHAP values) of specific immunometabolic indices to the model predictions. The SHAP values indicate how each index contributes positively or negatively towards the prediction of diabetic retinopathy. Colors represent values of indices as indicated by the scale bars: frailty score, HOMA-IR, HOMA-β, LS7 total score, and LAP (Lipid Accumulation Product).
