# Supplementary material for: Integration of Immunometabolic Composite Indices and Machine Learning for Diabetic Retinopathy Risk Stratification: Insights from NHANES 2011 – 2020
Source: Ophthalmol Sci. 2025 Jun 16;5(6):100854. doi: 10.1016/j.xops.2025.100854 (PMC12329596; doi:10.1016/j.xops.2025.100854)
Supplement: Figure S4 [file mmc15.pdf]

FigureS4

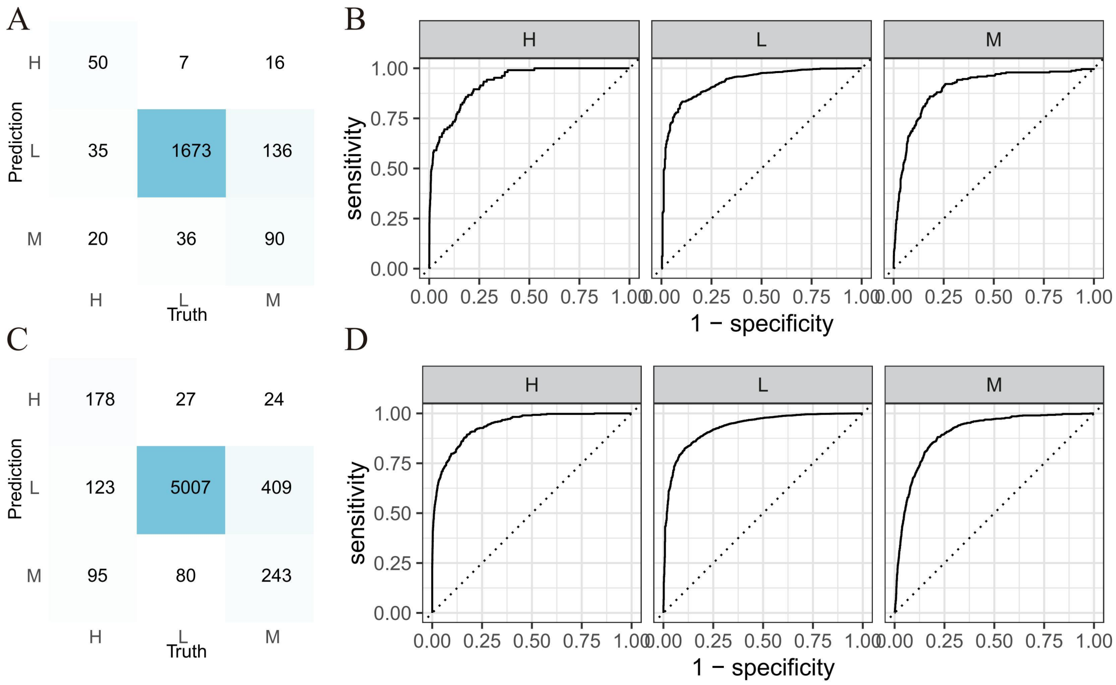

Figure S4: Model Validation Results

This figure presents confusion matrices and ROC curves for another validation dataset or an alternative model.

Panel (A, C): Confusion matrices showing classification accuracy.

Panel (B, D): ROC curves indicating model performance.
