# Supplementary material for: Integration of Immunometabolic Composite Indices and Machine Learning for Diabetic Retinopathy Risk Stratification: Insights from NHANES 2011 – 2020
Source: Ophthalmol Sci. 2025 Jun 16;5(6):100854. doi: 10.1016/j.xops.2025.100854 (PMC12329596; doi:10.1016/j.xops.2025.100854)
Supplement: Figure S8 [file mmc19.pdf]

FigureS8

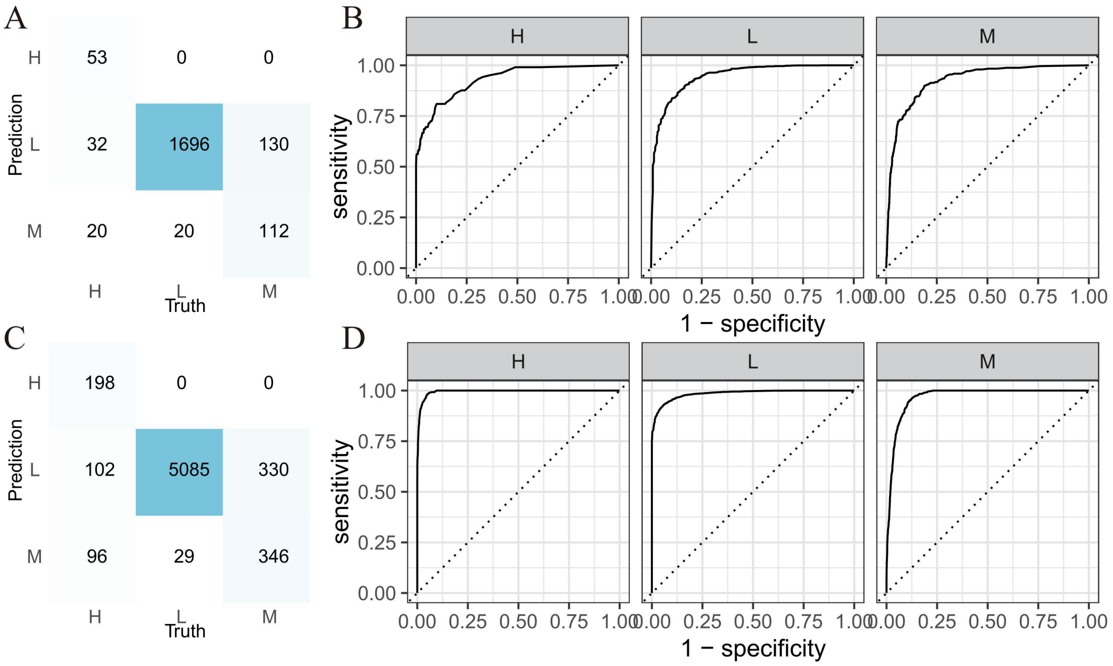

Figure S8: Model Performance Evaluation

This figure further evaluates the model performance using confusion matrices and ROC curves.

Panel (A, C): Confusion matrices displaying the model's predictions compared to ground truth.

Panel (B, D): ROC curves showing classification performance across different categories.
