# Supplementary material for: Integration of Immunometabolic Composite Indices and Machine Learning for Diabetic Retinopathy Risk Stratification: Insights from NHANES 2011 – 2020
Source: Ophthalmol Sci. 2025 Jun 16;5(6):100854. doi: 10.1016/j.xops.2025.100854 (PMC12329596; doi:10.1016/j.xops.2025.100854)
Supplement: Table S1 [file mmc21.pdf]

.metric	.estimator	.estimate	dataset	model
accuracy	multiclass	0.9076947[illegible]	train	xgboost
kap	multiclass	0.6355213[illegible]	train	xgboost
sens	macro	0.6702016[illegible]	train	xgboost
spec	macro	0.8567676[illegible]	train	xgboost
ppv	macro	0.8734225[illegible]	train	xgboost
npv	macro	0.9506690[illegible]	train	xgboost
mcc	multiclass	0.6567803[illegible]	train	xgboost
j_index	macro	0.5269692[illegible]	train	xgboost
bal_accura	macro	0.7634846[illegible]	train	xgboost
detection_	macro	0.3333333[illegible]	train	xgboost
precision	macro	0.8734225[illegible]	train	xgboost
recall	macro	0.6702016[illegible]	train	xgboost
f_meas	macro	0.74148511	train	xgboost
roc_auc	hand_till	0.9265844[illegible]	train	xgboost
accuracy	multiclass	0.9059621[illegible]	test	xgboost
kap	multiclass	0.6180406[illegible]	test	xgboost
sens	macro	0.66417471	test	xgboost
spec	macro	0.8494471[illegible]	test	xgboost
ppv	macro	0.8679765[illegible]	test	xgboost
npv	macro	0.9383061[illegible]	test	xgboost
mcc	multiclass	0.63816881	test	xgboost
j_index	macro	0.5136218[illegible]	test	xgboost
bal_accura	macro	0.7568109[illegible]	test	xgboost
detection_	macro	0.3333333[illegible]	test	xgboost
precision	macro	0.8679765[illegible]	test	xgboost
recall	macro	0.66417471	test	xgboost
f_meas	macro	0.7356529[illegible]	test	xgboost
roc_auc	hand_till	0.89573417	test	xgboost
